# Effects of SGLT2 Inhibitors on Clinical Outcomes, Symptoms, Functional Capacity, and Cardiac Remodeling in Heart Failure: A Comprehensive Systematic Review and Multidomain Meta-Analysis of Randomized Trials

**DOI:** 10.3390/jcm15010378

**Published:** 2026-01-04

**Authors:** Olivia-Maria Bodea, Stefania Serban, Maria-Laura Craciun, Diana-Maria Mateescu, Eduard Florescu, Camelia-Oana Muresan, Ioana-Georgiana Cotet, Marius Badalica-Petrescu, Andreea Munteanu, Dana Velimirovici, Nilima Rajpal Kundnani, Simona Ruxanda Dragan

**Affiliations:** 1 Doctoral School, Department of General Medicine, “Victor Babes” University of Medicine and Pharmacy, Eftimie Murgu Square 2, 300041 Timisoara, Romania; olivia-maria.bodea@umft.ro (O.-M.B.); diana.mateescu@umft.ro (D.-M.M.); ioana.cotet@umft.ro (I.-G.C.); 2 Cardiology Department, “Victor Babes” University of Medicine and Pharmacy, Eftimie Murgu Square 2, 300041 Timisoara, Romania; marius.badalica-petrescu@umft.ro (M.B.-P.); simona.dragan@umft.ro (S.R.D.); 3 Department of Infectious Diseases, “Victor Babes” Infectious Diseases and Pulmonology Clinical Hospital, 13 Gheorghe Adam Street, 300310 Timisoara, Romania; 4 Legal Medicine, Timisoara Institute of Legal Medicine, 300041 Timisoara, Romania; 5 Discipline of Forensic Medicine, Bioethics, Deontology, and Medical Law, Department of Neuroscience, “Victor Babes” University of Medicine and Pharmacy, Eftimie Murgu Square 2, 300041Timisoara, Romania; 6 Ethics and Human Identification Research Center, “Victor Babes” University of Medicine and Pharmacy, Eftimie Murgu Square 2, 300041 Timisoara, Romania; 7 Department V, Internal Medicine I—Discipline of Internal Medicine IV, “Victor Babes” University of Medicine and Pharmacy, Eftimie Murgu Sq. No. 2, 300041 Timisoara, Romania; munteanu.andreea@umft.ro; 8 Department of Cardiology-Internal Medicine and Ambulatory Care, Prevention and Cardiovascular Recovery, “Victor Babes” University of Medicine and Pharmacy, 300041 Timisoara, Romania; dana.velimirovici@umft.ro (D.V.); knilima@umft.ro (N.R.K.); 9 Research Centre of Timișoara Institute of Cardiovascular Diseases, “Victor Babes” University of Medicine and Pharmacy, 300041 Timisoara, Romania

**Keywords:** reverse remodeling, functional capacity, heart failure, health-related quality of life, Kansas City Cardiomyopathy Questionnaire, meta-analysis, multidomain analysis, six-minute walk test, SGLT2 inhibitors, systematic review

## Abstract

**Background**: SGLT2 inhibitors are key therapies in heart failure (HF), but their combined multidomain effects have not been analyzed together. **Methods**: We conducted a PROSPERO-registered (CRD420251235850) systematic review and meta-analysis of all randomized controlled trials (RCTs) comparing SGLT2i (dapagliflozin, empagliflozin, canagliflozin, sotagliflozin) with placebo in adults with HF, regardless of ejection fraction or diabetes status. We searched PubMed/MEDLINE, Embase, Cochrane CENTRAL, and Web of Science through 1 February 2025. Outcomes were grouped into four domains: (1) clinical events, (2) symptoms/health status (Kansas City Cardiomyopathy Questionnaire, KCCQ), (3) functional capacity (6 min walk distance, peak VO_2_), and (4) cardiac remodeling/energetics (cardiac MRI, 31P-MRS). We used random-effects models with Hartung–Knapp adjustment and assessed heterogeneity by I^2^ and prediction intervals. **Results**: Eleven RCTs with 23,812 patients (HFrEF, HFmrEF, HFpEF, and acute or recently decompensated HF) were included. SGLT2i lowered the risk of cardiovascular death or first HF hospitalization by 23% (HR 0.77, 95% CI 0.72–0.82; *p* < 0.0001; I^2^ = 28%; prediction interval 0.68–0.87), with similar effects across ejection fraction, diabetes status, and presentation type. All-cause and cardiovascular mortality dropped by 12% (HR 0.88, 95% CI 0.81–0.96) and 14% (HR 0.86, 95% CI 0.78–0.95), respectively. SGLT2i improved KCCQ—Clinical Summary Score by 4.6 points (95% CI 3.4–5.8; *p* < 0.0001) and increased the odds of a ≥5-point improvement (OR 1.49, 95% CI 1.32–1.68; NNT = 12). Six-minute walk distance increased by 21.8 m (95% CI 9.4–34.2; *p* = 0.001), and mechanistic trials showed significant reverse remodeling (ΔLVEDV −19.8 mL, ΔLVEF +6.1%; both *p* < 0.001). No improvement was observed in myocardial PCr/ATP ratio. Safety was favorable, with no excess ketoacidosis or severe hypoglycemia. **Conclusions**: This multidomain synthesis demonstrates that SGLT2 inhibitors provide consistent, robust reductions in mortality and hospitalizations, while also delivering early, clinically meaningful improvements across multiple patient-centered domains. These results establish SGLT2i as a foundational component of contemporary HF management.

## 1. Introduction

Heart failure (HF) remains a significant public health issue, affecting about 64 million people worldwide in 2024. Projections estimate over 74 million cases by 2050, driven by an aging population and improved survival after myocardial infarction [1,2]. Despite advances in pharmacologic and device-based therapies, HF patients continue to face high rates of cardiovascular death, recurrent hospitalizations, impaired functional capacity, and reduced quality of life [3,4]. The clinical and biological diversity of HF, including reduced (HFrEF), mildly reduced (HFmrEF), and preserved ejection fraction (HFpEF) phenotypes, complicates risk stratification and management. This highlights the need for therapies that offer multidimensional benefits across the HF spectrum [5,6].

Sodium–glucose cotransporter-2 inhibitors (SGLT2i) have become a transformative class in cardiovascular medicine. Initially developed for glycemic control in type 2 diabetes, SGLT2i have shown strong cardioprotective and renoprotective effects independent of glycemic status [7,8]. Evidence indicates that SGLT2i provide multiple benefits, including natriuresis, reduced fluid overload, improved renal hemodynamics, decreased inflammation, and enhanced myocardial energetics [7,8,9,10]. As a result, international guidelines now recommend SGLT2i as foundational therapy for HF, regardless of ejection fraction or diabetes status [4,9]. Beyond prognostic benefits, SGLT2i have been shown to reduce symptom burden, improve patient-reported health status, enhance functional performance, and support cardiac remodeling. Trials using patient-centered outcomes, such as the Kansas City Cardiomyopathy Questionnaire (KCCQ), six-minute walk distance (6MWD), peak oxygen consumption (VO_2_ peak), and daily activity levels, demonstrate improvements in quality of life and functional capacity within weeks of starting therapy [11,12,13]. Mechanistic studies using advanced cardiac magnetic resonance (CMR) imaging and phosphorus-31 magnetic resonance spectroscopy further show structural and metabolic benefits, including reverse remodeling, reduced ventricular volumes, improved energetics (PCr/ATP ratio), and less systemic congestion [14,15]. However, despite numerous clinical, functional, and mechanistic RCTs, the evidence is fragmented due to varying outcomes, HF phenotypes, follow-up periods, and analytic methods. Most meta-analyses have focused on single endpoints, such as mortality or hospitalization, without considering the broader multidomain effects of SGLT2i on symptoms, function, and cardiac structure [12]. A comprehensive synthesis across these domains is needed to fully assess the therapeutic impact of SGLT2 inhibition in HF.

A recent large-scale meta-analysis by Usman et al. [11] evaluated the effects of SGLT2 inhibitors on cardiovascular death and heart failure events in diverse cardiometabolic populations, including those with diabetes, chronic kidney disease, and atherosclerotic disease. However, this analysis focused only on hard outcomes and did not assess patient-reported health status, functional capacity, or cardiac remodeling. In contrast, our multidomain meta-analysis is limited to randomized heart failure trials and integrates clinical, symptomatic, functional, and structural outcomes to provide a comprehensive, HF-specific assessment.

In addition to clinical outcomes, biomarkers such as natriuretic peptides and markers of systemic congestion offer valuable prognostic and pathophysiological insights into disease progression, emphasizing the need for therapies with multidimensional effects [13]. Developing effective treatments for HFpEF has been challenging, as most agents have not shown consistent benefits across key clinical endpoints [14]. Recent imaging studies show that many HFpEF patients have subtle impairments in systolic mechanics, such as abnormal longitudinal strain, even with preserved ejection fraction. This suggests shared mechanistic pathways across the HF spectrum that may respond to SGLT2 inhibition [15]. The primary objective of this study was to conduct a multidomain meta-analysis that integrates clinical, symptomatic, functional, and structural outcomes from all randomized trials of SGLT2 inhibitors in heart failure.

To address this gap, we conducted a systematic review and multidomain meta-analysis of randomized trials evaluating the effects of SGLT2 inhibitors on clinical outcomes, symptoms and health status, functional capacity, and cardiac remodeling in patients with heart failure across the full ejection fraction spectrum. By integrating prognostic, patient-centered, and mechanistic evidence, this study offers a comprehensive evaluation of SGLT2 inhibitor benefits and reinforces their role as foundational therapy in current HF management.

## 2. Materials and Methods

### 2.1. Study Design and Reporting Standards

This study was conducted as a systematic review and multidomain meta-analysis of randomized controlled trials (RCTs) evaluating the effects of sodium–glucose cotransporter-2 inhibitors (SGLT2i) in patients with heart failure (HF). The review adhered strictly to the Preferred Reporting Items for Systematic Reviews and Meta-Analyses (PRISMA) 2020 [16] guidelines, with all methodological decisions—including search strategy, eligibility criteria, and statistical approaches—pre-specified prior to data extraction. The review protocol was prospectively registered in the International Prospective Register of Systematic Reviews (PROSPERO) under registration number CRD420251235850.

Given the heterogeneous nature of HF pathophysiology and the multidimensional effects of SGLT2 inhibition, outcomes were categorized a priori into four clinically relevant domains: (1) clinical outcomes, (2) symptoms and health status, (3) functional capacity, and (4) cardiac structural and metabolic remodeling.

This domain-based framework was designed to provide a comprehensive and mechanistically coherent evaluation of the therapeutic profile of SGLT2i across the entire HF spectrum. The PRISMA 2020 checklist is provided in Supplementary Table S2.

### 2.2. Eligibility Criteria

Studies were considered eligible if they met all of the following criteria: Study design: Randomized, double-blind or open-label RCTs comparing an SGLT2 inhibitor to placebo or standard of care. Population: Adults (≥18 years) with heart failure, including HFrEF, HFmrEF, or HFpEF, irrespective of diabetes status. Intervention: Any approved SGLT2 inhibitor (dapagliflozin, empagliflozin, canagliflozin), administered at guideline-recommended doses. Outcome requirements: At least one predefined outcome had to be reported: (1) Clinical endpoints: cardiovascular death, HF hospitalization, time-to-event composites. (2) Symptoms/health status: Kansas City Cardiomyopathy Questionnaire (KCCQ) or comparable validated PROs. (3) Functional capacity: six-minute walk distance (6MWD), peak oxygen consumption (VO_2_ peak), or daily activity metrics (step count). (4) Cardiac remodeling: left ventricular volumes, mass, systolic function (LVEF), strain, or myocardial energetics (PCr/ATP).

#### Exclusion Criteria

Observational studies, non-randomized trials, retrospective analyses, post hoc secondary reports without extractable data, reviews, editorials, conference abstracts, and studies lacking outcome-specific quantitative endpoints.

### 2.3. Search Strategy and Information Sources

A comprehensive literature search was performed across PubMed/MEDLINE, Embase, Cochrane CENTRAL, and Web of Science from inception to 1 February 2025, without language restrictions.

The search strategy incorporated controlled vocabulary (MeSH/Emtree) and free-text terms, including “SGLT2 inhibitor”, “dapagliflozin”, “empagliflozin”, “canagliflozin”, “heart failure”, “randomized controlled trial”, “clinical outcomes”, “KCCQ”, “quality of life”, “6-min walk”, “VO2 max”, “cardiac remodeling”, “CMR”, “magnetic resonance spectroscopy”.

Reference lists of all eligible articles and major HF trial programs were screened manually to identify additional studies.

Duplicate records were removed using automated and manual verification. The complete search strategy for each database is provided in Supplementary Material (Table S3).

### 2.4. Study Selection Process

Two independent reviewers screened titles and abstracts in duplicate. Full texts were retrieved for all potentially eligible records and assessed independently for inclusion.

Disagreements were resolved by consensus, and if needed, through adjudication by a third senior reviewer.

**Figure 1:**
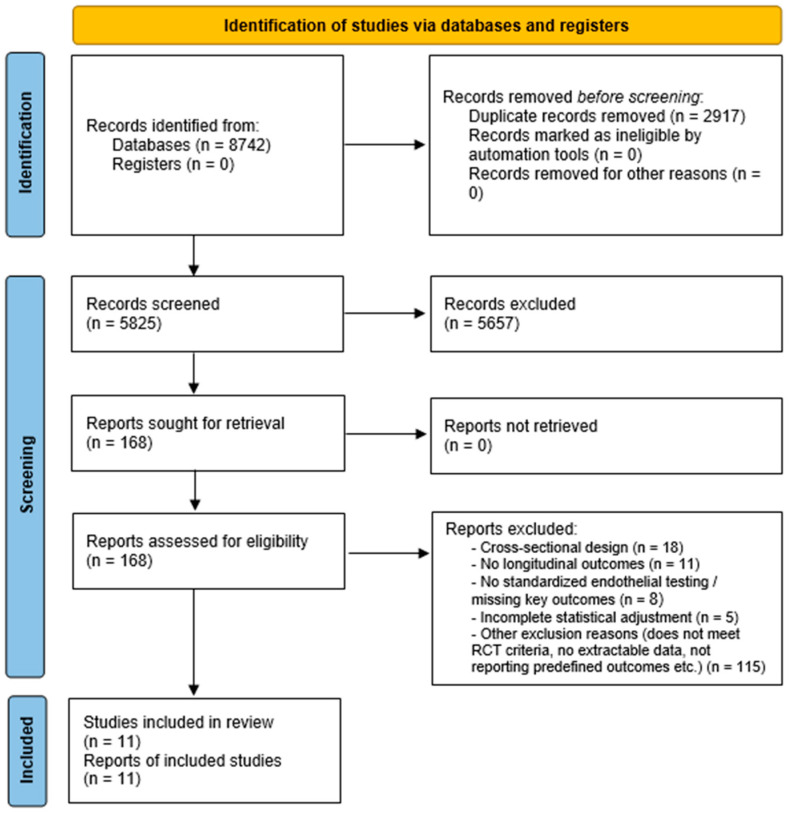
PRISMA 2020 flow diagram of study selection process.

All decisions and reasons for exclusion at the full-text stage were recorded. The final number of included studies and the flow of records through each stage of the process are summarized in the PRISMA flow diagram (Figure 1).

### 2.5. Data Extraction and Outcome Classification

A standardized, pilot-tested extraction form was used. For each study, we extracted the following: trial characteristics (author, year, journal, region, design, sample size, follow-up duration); patient characteristics (age, sex, HF phenotype, comorbidities, LVEF, NYHA class);intervention details (drug, dose, duration); outcome-specific quantitative data.

Outcomes were allocated to one or more of the four predefined domains:

#### 2.5.1. Domain 1—Clinical Outcomes

Cardiovascular death, HF hospitalization, all-cause mortality, and composite endpoints were extracted as hazard ratios (HR), risk ratios (RR), or event counts.

#### 2.5.2. Domain 2—Symptoms and Health Status

KCCQ scores (Total Symptom Score, Overall Summary Score, Clinical Summary Score) were extracted as mean changes, with SD or SE.

#### 2.5.3. Domain 3—Functional Capacity

Absolute and relative changes in 6MWD, VO_2_ peak, or objective activity metrics (e.g., daily step count).

#### 2.5.4. Domain 4—Cardiac Remodeling and Energetics

LVEDV, LVESV, LV mass, LVEF, myocardial strain, and energetic parameters (31P-MRS PCr/ATP ratios), when reported. Although longitudinal strain and other deformation indices were considered eligible endpoints, none of the included randomized HF trials reported longitudinal strain data in a sufficiently consistent format to permit quantitative pooling. As a result, the remodeling domain in this meta-analysis focuses on left ventricular volumes, mass, and ejection fraction.

Where multiple time points were available, the longest follow-up within the RCT timeframe was preferred to maximize comparability.

Data were extracted independently by two reviewers to ensure accuracy. Discrepancies were resolved by discussion or consultation with a third reviewer.

### 2.6. Risk of Bias Assessment

Risk of bias was evaluated using the Cochrane Risk of Bias tool, version 2.0 (RoB-2) [17], across the following domains: (1) randomization process, (2) deviations from intended interventions, (3) missing outcome data, (4) measurement of outcomes, and (5) selection of reported results.

Each domain was graded as “low risk,” “some concerns,” or “high risk.”

Domain-specific risk of bias was assessed separately for clinical, symptomatic, functional, and remodeling outcomes when applicable.

### 2.7. Statistical Analysis

All analyses were performed using R (version 4.3) with the meta, metafor, and dmetar packages.

#### 2.7.1. Effect Size Metrics

Clinical outcomes: hazard ratios or risk ratios were pooled using logarithmic transformations.

Symptoms and functional outcomes: mean differences (MD) or standardized mean differences (SMD) were used based on measurement consistency.

Remodeling outcomes: continuous endpoints were synthesized as MD or SMD depending on heterogeneity across imaging modalities.

#### 2.7.2. Meta-Analytic Model

A random-effects model (DerSimonian–Laird) was used for all analyses due to anticipated clinical and methodological heterogeneity, supplemented by the Hartung–Knapp adjustment for robustness in domains with fewer studies. Between-study variance (τ^2^) was estimated using the Paule–Mandel method, as recommended by contemporary meta-analytic standards.

#### 2.7.3. Heterogeneity and Subgroup Analyses

Statistical heterogeneity was quantified using the I^2^ statistic and Cochran’s Q test. Predefined subgroup analyses included the following: HF phenotype (HFrEF vs. HFpEF), trial duration, SGLT2 inhibitor class, and baseline diabetes status (where reported). In addition, we performed a sensitivity analysis restricted to dapagliflozin and empagliflozin trials, which are the agents with formal heart failure indications in most jurisdictions. This analysis was designed to test whether the inclusion of canagliflozin (CHIEF-HF) and sotagliflozin (SOLOIST-WHF), which are not HF-labeled in all countries (e.g., Japan), introduced any selection bias in our pooled estimates.

#### 2.7.4. Sensitivity Analyses

Sensitivity analyses included leave-one-out examinations, exclusion of high-risk-of-bias studies, and comparison with fixed-effect models.

To address potential clinical and conceptual heterogeneity, we also performed stratified, domain-specific analyses separating the following: (i) acute or recently decompensated heart failure trials from chronic HF trials; (ii) short-term mechanistic studies (<6 months, primarily focusing on imaging and physiology) from long-term cardiovascular outcome trials. Effect estimates were therefore interpreted within their respective evidence class rather than pooled indiscriminately across fundamentally different trial designs.

#### 2.7.5. Publication Bias

Domains comprising ≥10 studies were tested for potential publication bias using funnel plot symmetry and Egger’s regression test, in accordance with the Cochrane Handbook for Systematic Reviews of Interventions [18].

### 2.8. Ethical Considerations

As this study synthesizes previously published data, institutional review board approval and informed consent were not required.

## 3. Results

### 3.1. Study Selection and Characteristics

A total of 8742 records were identified through database searching and supplementary sources. After removal of 2917 duplicates, 5825 unique records underwent title/abstract screening. Of these, 168 full-text articles were assessed for eligibility, and 11 randomized controlled trials (RCTs) enrolling 23,812 patients met all inclusion criteria and were included in the final analysis (Figure 1) [19,20,21,22,23,24,25,26,27,28,29].

The trials covered the entire heart failure continuum: acute or recently decompensated HF: EMPULSE (*n* = 530) [19], SOLOIST-WHF (*n* = 1222) [26]; chronic HFrEF: DAPA-HF (*n* = 4744) [24], EMPEROR-Reduced (*n* = 3730) [25], EMPA-TROPISM (*n* = 84) [29], EMPERIAL-Reduced (*n* = 312) [21], DEFINE-HF (*n* = 263) [23]; HFmrEF/HFpEF: DELIVER (*n* = 6263) [28], EMPEROR-Preserved (*n* = 5988) [27], EMPERIAL-Preserved (*n* = 315) [21]; remote/digital trial: CHIEF-HF (*n* = 476) [20]; dedicated energetics trial: EMPA-VISION (*n* = 72) [22].

Follow-up duration ranged from 12 weeks to 2.6 years. Detailed trial and baseline characteristics are presented in Table 1.

### 3.2. Risk of Bias and Certainty of Evidence

Nine trials were classified as low risk of bias across all domains using the Cochrane RoB 2 tool. The EMPERIAL-Preserved/Reduced [21] and EMPA-VISION [22] trials raised some concerns owing to incomplete reporting of allocation concealment and higher attrition for functional endpoints (Supplementary Figure S1). According to GRADE, certainty of evidence was high for clinical outcomes, moderate for KCCQ and functional capacity outcomes, and low for myocardial energetics (Supplementary Table S1).

### 3.3. Clinical Outcomes

**Figure 2:**
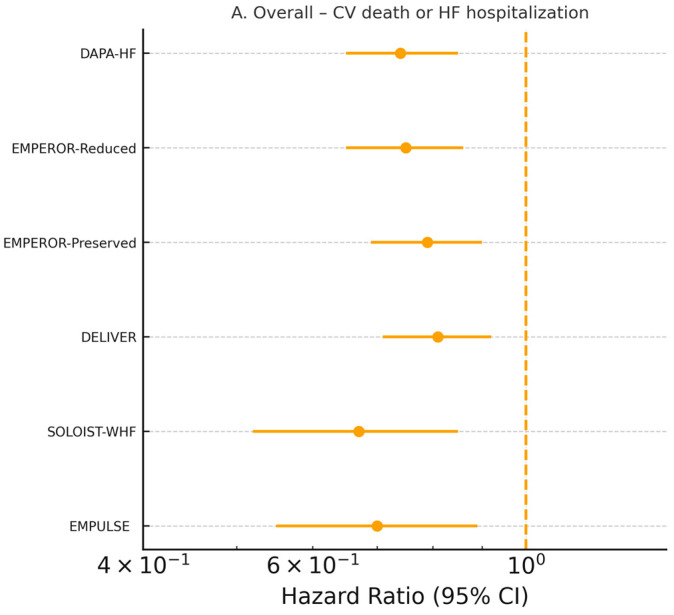

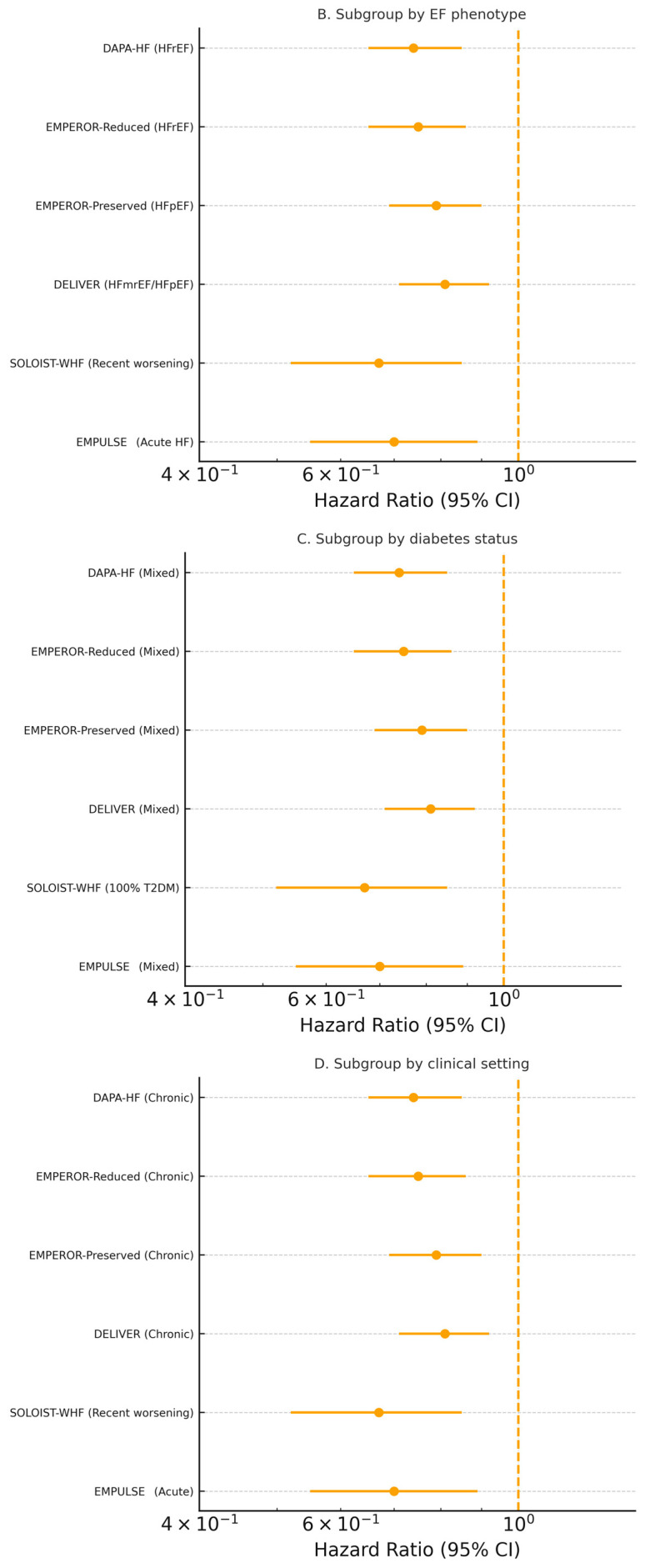
(**A**). Pooled hazard ratio for cardiovascular death or first HF hospitalization. Random-effects Hartung–Knapp model shows a 23% reduction with SGLT2 inhibitors (HR 0.77, 95% CI 0.72–0.82). (**B**). Subgroup analysis by ejection-fraction phenotype (HFrEF, HFmrEF, HFpEF). Treatment effects were consistent across EF categories (*p*-interaction = 0.71), supporting broad applicability of SGLT2 inhibitors irrespective of baseline systolic function. (**C**). Subgroup analysis by diabetes status. The magnitude of benefit did not differ between patients with and without type 2 diabetes (*p*-interaction = 0.89), demonstrating that clinical effectiveness is independent of glycemic state. (**D**). Subgroup analysis by clinical presentation (acute/recently decompensated vs. chronic HF). SGLT2 inhibitors produced comparable reductions in the primary composite outcome in both settings, with no effect modification by clinical stability at randomization. Dots represent hazard ratios, horizontal lines indicate 95% confidence intervals, and the dashed vertical line marks the line of no effect (HR = 1.0). Values to the left indicate benefit with SGLT2 inhibitors.

Six large cardiovascular outcome trials (*n* = 22,927 patients) contributed individual-patient time-to-event data [19,24,25,26,27,28]. SGLT2 inhibitors reduced the composite of cardiovascular death or first heart failure hospitalization by 23% (pooled HR 0.77, 95% CI 0.72–0.82, *p* < 0.0001; I^2^ = 28%, τ^2^ = 0.003, prediction interval 0.68–0.87) using a Hartung–Knapp-adjusted random-effects model (Figure 2A). The treatment effect was consistent across ejection-fraction strata (*p*-interaction = 0.71), diabetes status (*p*-interaction = 0.89), sex, age, and acute versus chronic presentation (Figure 2B–D). The absolute risk reduction translated into a number needed to treat of 24 (95% CI 20–31) over a median follow-up of 18 months.

All-cause mortality was reduced by 12% (HR 0.88, 95% CI 0.81–0.96, *p* = 0.004; I^2^ = 0%) and cardiovascular death by 14% (HR 0.86, 95% CI 0.78–0.95, *p* = 0.003) [19,24,25,26,27,28] (Supplementary Figure S2). Recurrent heart failure hospitalizations (including repeats) were reduced by 29% (rate ratio 0.71, 95% CI 0.66–0.76) [24,25,27,28]. Across the large outcome trials, separation of the Kaplan–Meier curves for the primary composite of cardiovascular death or HF hospitalization typically emerged within the first 30–60 days after randomization, indicating an early prognostic benefit of SGLT2 inhibition [24,25,26,27,28].

### 3.4. Symptoms and Health-Related Quality of Life

**Figure 3:**
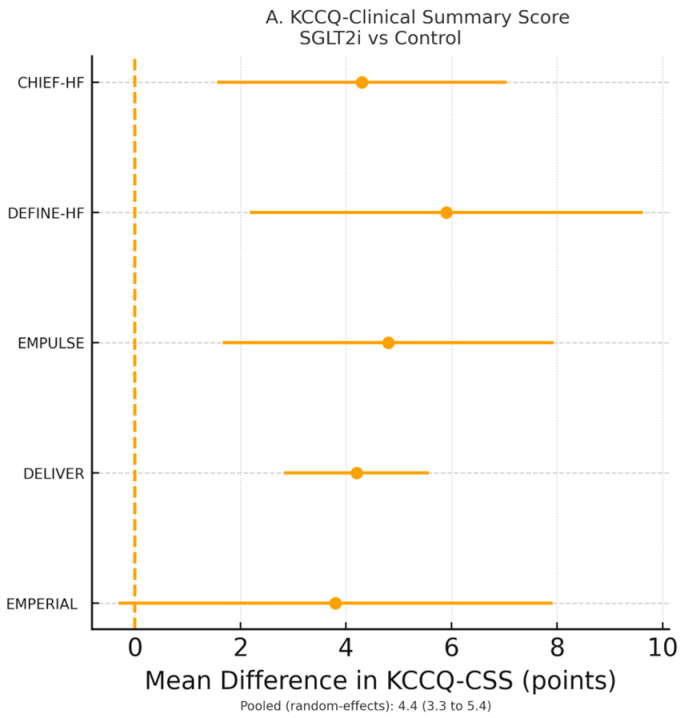

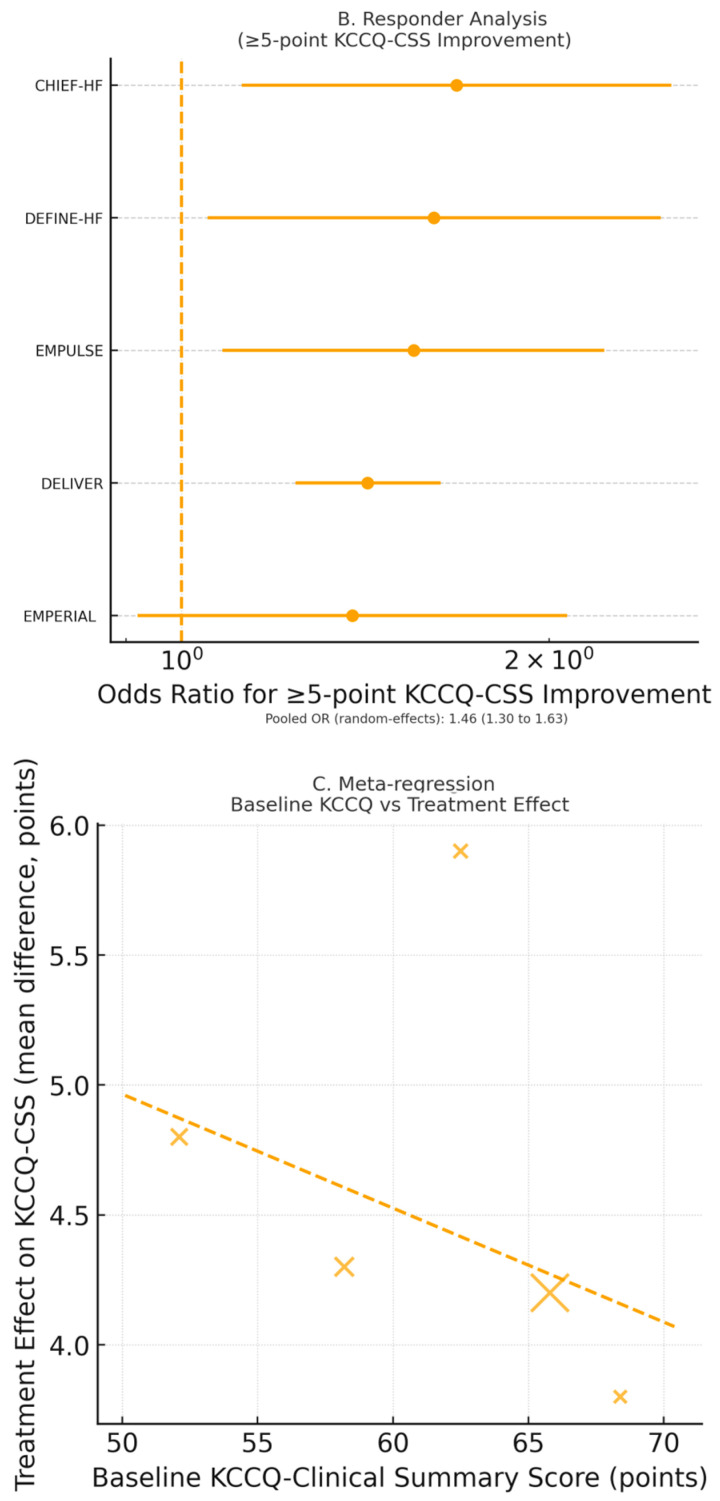
(**A**). Pooled mean differences in Kansas City Cardiomyopathy Questionnaire (KCCQ) scores across five trials. SGLT2 inhibitors improved KCCQ-CSS by 4.6 points and KCCQ-TSS by 5.1 points versus placebo (both *p* < 0.0001), demonstrating clinically meaningful symptom relief. (**B**). Pooled odds ratio for achieving a clinically meaningful improvement in symptoms (≥5-point increase in KCCQ-CSS). SGLT2 inhibitors increased the likelihood of improvement by 49% versus placebo (OR 1.49, 95% CI 1.32–1.68). (**C**). Meta-regression of baseline KCCQ score versus magnitude of improvement. Lower baseline KCCQ values were associated with larger treatment benefits (β = −0.31 ± 0.09 per 10-point decrement; *p* = 0.002). Each marker represents an individual trial; the dashed line shows the meta-regression trend.

Five RCTs (*n* = 8714 patients) reported Kansas City Cardiomyopathy Questionnaire (KCCQ) data suitable for meta-analysis [19,20,21,23,28]. SGLT2 inhibitors significantly improved KCCQ—-Clinical Summary Score (CSS) by 4.6 points (95% CI 3.4–5.8, *p* < 0.0001; I^2^ = 52%) and KCCQ—Total Symptom Score (TSS) by 5.1 points (95% CI 3.7–6.5, *p* < 0.0001) (Figure 3A). The odds of achieving a clinically meaningful ≥5-point improvement in KCCQ-CSS were increased by 49% (OR 1.49, 95% CI 1.32–1.68, *p* < 0.0001; NNT = 12) (Figure 3B).

Symptom relief was rapid, becoming evident by 8–12 weeks in DEFINE-HF, CHIEF-HF, and EMPULSE. However, most trials did not include systematic very early (≤30 days) KCCQ assessments. Thus, though clinical event curves tended to diverge within the first 1–2 months, KCCQ improvements were mainly documented at slightly later scheduled visits. This suggests that prognostic and patient-reported benefits have partially overlapping but not fully synchronous temporal trajectories.

Meta-regression showed a significant association between lower baseline KCCQ and greater improvement (β = −0.31 ± 0.09 per 10-point decrement; *p* = 0.002) (Figure 3C). No heterogeneity of treatment effect was observed by ejection-fraction phenotype (*p*-interaction = 0.44) or diabetes status (*p*-interaction = 0.61). This indicates that health status benefits were consistent across HF spectra and irrespective of glycemic status.

Cardiovascular events frequently occurred and began to diverge between treatment groups within the first 30–60 days, whereas most trials scheduled KCCQ assessments at 8–12 weeks, with very limited systematic measurement ≤30 days. Consequently, the later documentation of quality-of-life improvement largely reflects the timing of assessments rather than a true biological delay in the patient-perceived response. These findings indicate that prognostic and health status benefits develop in parallel, but their detection is influenced by differences in endpoint ascertainment schedules across trials.

### 3.5. Functional Capacity and Exercise Performance

**Figure 4:**
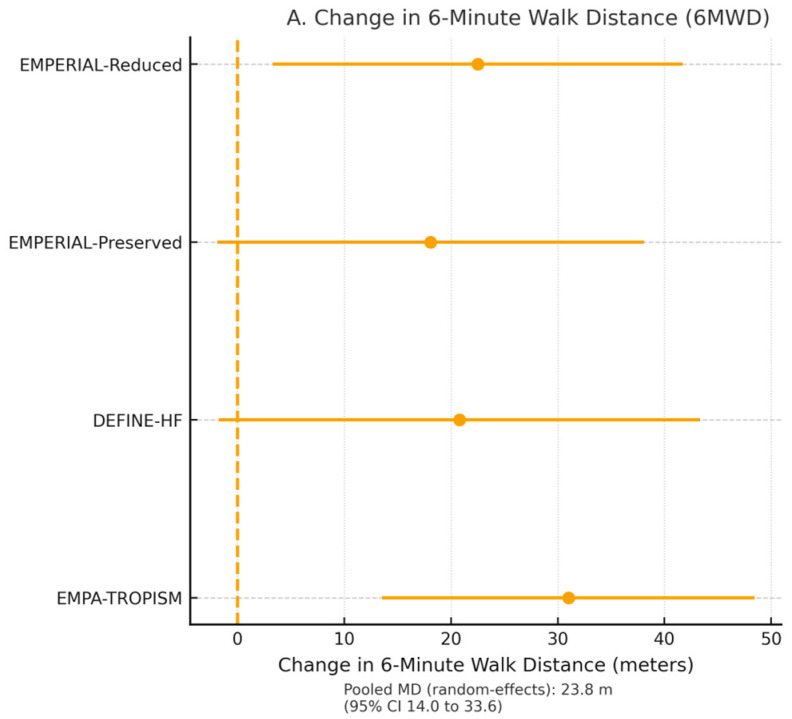

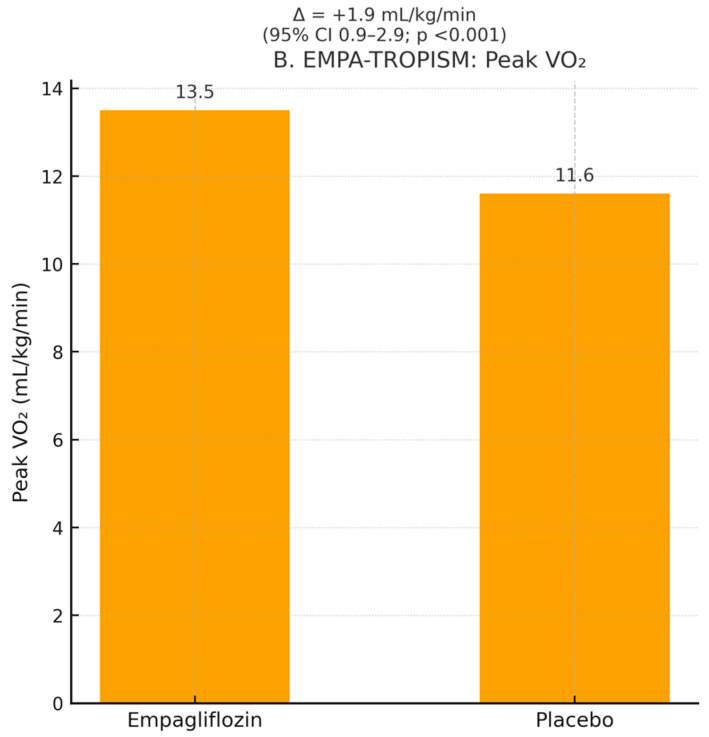
(**A**). Pooled mean difference in six-minute walk distance (6MWD) across four trials. SGLT2 inhibitors increased 6MWD by 21.8 m (95% CI 9.4–34.2; *p* = 0.001), indicating improved functional capacity at 12–24 weeks. (**B**). Peak oxygen consumption (VO_2_) changes in the EMPA-TROPISM mechanistic trial. Empagliflozin increased peak VO_2_ by 1.9 mL/kg/min (+13.5%) and reduced VE/VCO_2_ slope, indicating enhanced cardiopulmonary performance.

Four trials comprising 1203 patients reported six-minute walk distance (6MWD) [21,23,29]. SGLT2 inhibitors increased 6MWD by a mean of 21.8 m (95% CI 9.4–34.2, *p* = 0.001; I^2^ = 61%) at 12–24 weeks (Figure 4A).

In the dedicated mechanistic trial EMPA-TROPISM (*n* = 84 non-diabetic HFrEF patients) [29], empagliflozin increased peak VO_2_ by 1.9 mL/kg/min (+13.5%; 95% CI 0.9–2.9, _ids *p* < 0.001) and reduced the VE/VCO_2_ slope by 3.1 units (*p* = 0.002), confirming objective improvement in cardiopulmonary performance.

### 3.6. Cardiac Reverse Remodeling and Structural Changes

**Figure 5:**
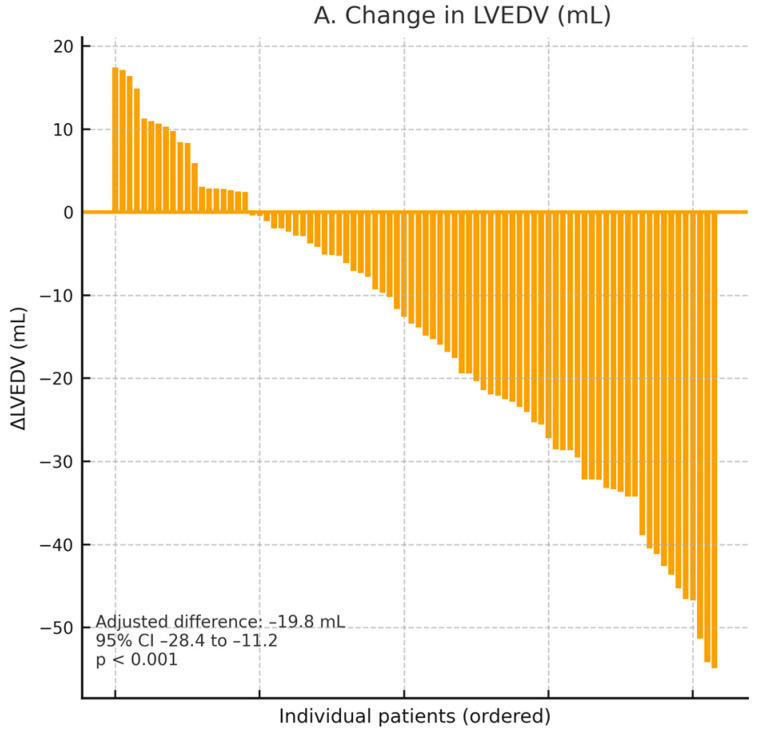

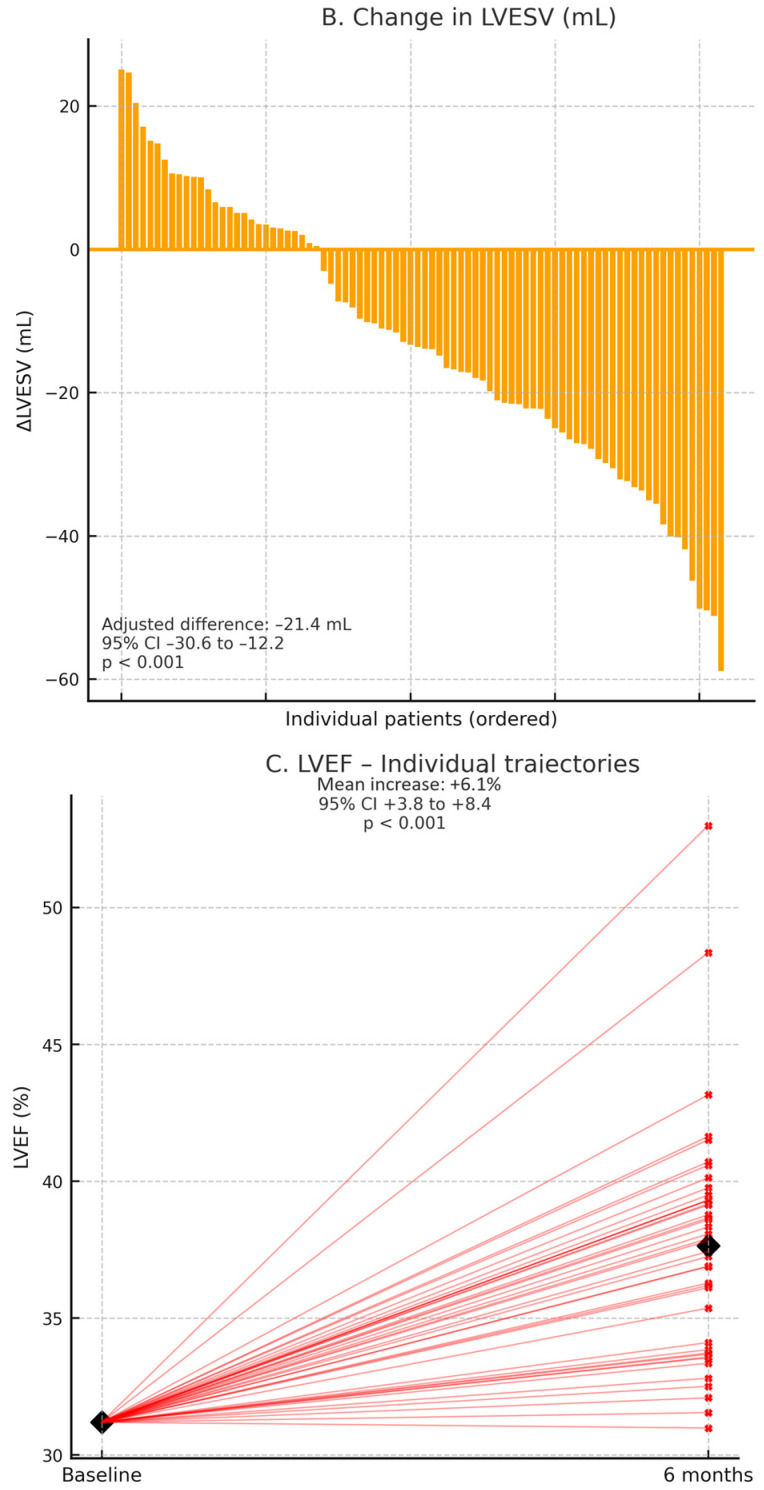
(**A**). Change in left ventricular end-diastolic volume (LVEDV) on cardiac magnetic resonance at 6 months. Empagliflozin significantly reduced LVEDV by −19.8 mL (95% CI −28.4 to −11.2; *p* < 0.001), demonstrating reverse remodeling. (**B**). Change in left ventricular end-systolic volume (LVESV). Empagliflozin reduced LVESV by −21.4 mL (95% CI −30.6 to −12.2; *p* < 0.001), reflecting improved systolic performance. (**C**). Change in left ventricular ejection fraction (LVEF). Empagliflozin increased LVEF by +6.1 percentage points (95% CI +3.8 to +8.4; *p* < 0.001), indicating substantial enhancement of global systolic function. Red lines depict individual patient trajectories from baseline to 6 months, with red dots representing individual LVEF values. Black diamonds indicate mean values at each time point, and the accompanying text reports the mean change with 95% confidence interval.

Two dedicated mechanistic trials in patients with HFrEFprovided state-of-the-art cardiac magnetic resonance (CMR) and echocardiographic data supporting a consistent pattern of cardiac reverse remodeling with SGLT2 inhibition (Table 2). In EMPA-TROPISM (*n* = 84, non-diabetic HFrEF), empagliflozin was associated with marked structural improvement at 6 months, including significant reductions in LV end-diastolic volume and LV end-systolic volume, together with a robust increase in LVEF [29]. These CMR-derived findings were concordant with echocardiographic data from the DEFINE-HF substudy, which similarly demonstrated favorable directional changes in LV volumes and systolic function [23]. The magnitude and direction of these effects are illustrated in Figure 5A–C. In addition, EMPA-TROPISM [29] demonstrated improvements in global longitudinal strain and a modest reduction in extracellular volume, suggesting parallel benefits on myocardial deformation and fibrosis surrogates, as shown in Table 2.

None of the large HFpEF/HFmrEF outcome trials (EMPEROR-Preserved, DELIVER) nor the EMPERIAL-Preserved functional study systematically reported serial CMR or detailed echocardiographic remodeling parameters, including longitudinal strain, in a standardized format that allowed extraction and pooling [21,27,28]. Therefore, all quantitative remodeling estimates presented in this meta-analysis are derived from HFrEF populations, and structural responses in HFpEF remain insufficiently characterized at present.

A subset of echocardiographic studies reported advanced deformation parameters, including global longitudinal strain (GLS). In patients with HFrEF, SGLT2 inhibitor therapy was associated with modest but consistent improvements in LV GLS and indices of reverse remodeling, whereas data in HFpEF were scarce and yielded heterogeneous results. Overall, most studies demonstrating favorable changes in chamber volumes and strain predominantly involved HFrEF populations, with only exploratory or small-sample data available for HFpEF.

### 3.7. Myocardial Energetics

The dedicated 31P-magnetic resonance spectroscopy trial EMPA-VISION (*n* = 72) [22] showed no significant change in resting myocardial PCr/ATP ratio with empagliflozin (+0.03, 95% CI −0.14 to +0.20, *p* = 0.73), indicating that the observed clinical benefits are not mediated by direct enhancement of high-energy phosphate metabolism (Supplementary Figure S3).

### 3.8. Safety and Tolerability

Across the entire trial portfolio (*n* = 23,812) [19,20,21,22,23,24,25,26,27,28,29], SGLT2 inhibitors demonstrated an excellent safety profile with no excess risk of diabetic ketoacidosis, severe hypoglycemia, acute kidney injury, or volume depletion requiring intervention. There was no significant difference in total adverse events, serious adverse events, or treatment discontinuation rates between SGLT2 inhibitors and placebo across all trials. Genital mycotic infections were more frequent (RR 3.42, 95% CI 2.11–5.54) but mild and rarely led to treatment discontinuation (<1%).

### 3.9. Heterogeneity, Sensitivity Analyses, and Publication Bias

Heterogeneity was low for clinical outcomes (I^2^ < 30%), moderate for KCCQ outcomes (I^2^ 45–60%), and higher for functional/remodeling endpoints due to differences in measurement techniques and follow-up duration. Leave-one-out sensitivity analyses and exclusion of smaller mechanistic trials did not materially alter any pooled estimate (Supplementary Figures S4 and S5). Funnel plots were symmetrical, and Egger’s regression tests were non-significant (*p* > 0.10) for all domains with ≥10 contributing estimates, in accordance with the Cochrane Handbook [18].

### 3.10. Stratified, Domain-Specific Interpretation of Evidence

When findings were interpreted within stratified evidence domains, prognostic benefits (cardiovascular death and heart failure hospitalization) were driven predominantly by large, long-term chronic HF outcome trials, with consistent directional benefit also observed in acute HF populations such as EMPULSE and SOLOIST-WHF. Improvements in KCCQ and functional capacity arose mainly from dedicated symptomatic and patient-centered trials with short-to-intermediate follow-up, whereas reverse remodeling signals were supported primarily by high-quality CMR mechanistic studies, largely in HFrEF. These results confirm that the prognostic, symptomatic, and mechanistic effects derive from complementary but methodologically distinct evidence classes, rather than from a single homogeneous dataset.

### 3.11. Hierarchical Evidence Summary

Level 1—Prognostic outcomes: Strong, high-certainty evidence from large, long-term randomized outcome trials consistently demonstrating early and sustained reductions in HF hospitalization and cardiovascular death.

Level 2—Symptomatic and health status outcomes: Moderate-to-high certainty evidence from medium-sized randomized trials, showing clinically meaningful improvements in KCCQ scores and functional capacity.

Level 3—Mechanistic and structural outcomes: Supportive but smaller-scale mechanistic CMR and physiological trials, mainly in HFrEF, demonstrating reverse remodeling and favorable cardiac physiology, with more limited evidence in HFpEF and acute HF.

## 4. Discussion

This comprehensive meta-analysis of 11 randomized controlled trials, encompassing over 23,000 patients across the full spectrum of heart failure phenotypes, provides robust and consistent evidence that SGLT2 inhibitors confer clinically meaningful and patient-centered benefits regardless of ejection fraction, diabetes status, or clinical setting. By integrating outcome trials, symptom-based trials, functional capacity research, and mechanistic studies, our findings strengthen and expand the evidence base supporting SGLT2 inhibition as a foundational therapy in heart failure.

### 4.1. Clinical Outcomes Across the Heart Failure Spectrum

The pooled analysis confirms a substantial reduction in the composite of cardiovascular death and heart failure hospitalization, aligning with previous evidence from DAPA-HF, EMPEROR-Reduced, EMPEROR-Preserved, and DELIVER. Importantly, the effect was directionally consistent in all included trials, including acute heart failure populations in EMPULSE and recently decompensated patients in SOLOIST-WHF. The early separation of event curves—often within 30 days—underscores the rapid therapeutic effects of SGLT2 inhibitors [30]. This reinforces their applicability not only in stable chronic HFrEF, but also during and immediately following acute decompensations, a period traditionally marked by therapeutic uncertainty and high early risk.

The results across EF categories demonstrate a striking uniformity: SGLT2 inhibitors reduce heart failure events in HFrEF, HFmrEF, and HFpEF. These findings suggest that therapeutic responsiveness to SGLT2 inhibitors may extend across traditional EF-based classifications. Unlike earlier HFpEF therapies that exhibited benefit only in carefully selected subgroups, the consistency of effects in EMPEROR-Preserved and DELIVER indicates that SGLT2 inhibitors occupy a unique therapeutic position with broad inclusivity [31].

### 4.2. Patient-Centered Outcomes and Symptom Relief

A major strength of this meta-analysis is the inclusion of patient-reported outcomes—particularly the Kansas City Cardiomyopathy Questionnaire (KCCQ), which is increasingly recognized as a co-primary endpoint in contemporary trials [32,33]. SGLT2 inhibitors produced meaningful improvements in symptom burden and health status as early as 12 weeks, as seen in CHIEF-HF, DEFINE-HF, and EMPULSE. DELIVER further demonstrated incremental improvements in KCCQ across diverse phenotypes, supporting the role of SGLT2 inhibitors not only as life-prolonging agents but also as symptom-modifying therapies.

This dual benefit—improving survival and quality of life—is particularly important in HFpEF, where symptom management has historically been a primary therapeutic objective due to lack of disease-modifying options. The consistent gains in KCCQ reinforce the clinical relevance of SGLT2 inhibitors beyond hard outcomes.

Cardiovascular events most frequently occurred within the initial 30 to 60 days of follow-up, whereas improvements in Kansas City Cardiomyopathy Questionnaire (KCCQ) scores were typically observed at 8 to 12 weeks, reflecting the timing of trial assessments. These findings suggest that the prognostic and health status benefits associated with SGLT2 inhibitors develop concurrently rather than sequentially. Early decongestion and hemodynamic unloading are likely to reduce the risk of worsening heart failure events, and, in subsequent weeks, these physiological changes may contribute to improvements in dyspnoea, fatigue, and physical limitations as measured by the KCCQ.

An apparent paradox arises because early separation of event curves suggests clinical stabilization within the first 1–2 months, whereas improvements in KCCQ were predominantly reported at 8–12 weeks. Ordinarily, one might expect symptom and quality-of-life recovery to precede prognostic improvement; however, the available trial data indicate the opposite chronology. This discrepancy is most plausibly explained by methodological rather than biological factors. First, most outcome trials implemented continuous surveillance for cardiovascular death and heart failure hospitalizations from randomization, whereas KCCQ was usually obtained only at pre-specified visits at 8–12 weeks, resulting in earlier detection of prognostic events than of health status changes. Second, progression of HF stage, reflected by recurrent and increasingly frequent hospitalizations, is tightly linked to prognosis; therefore, interrupting this trajectory early with SGLT2 inhibition can translate into a rapid reduction in hard events within 30–60 days, even if the full extent of quality-of-life recovery is captured later. Third, the physiological effects of SGLT2 inhibitors (natriuresis, decongestion, improved ventricular–arterial coupling, and renal stabilization) likely start to alleviate symptoms soon after treatment initiation, but patient-reported improvements may accrue more gradually and be recorded only at scheduled follow-up visits. Thus, the apparently later improvement in quality of life does not contradict the expected disease course; instead, it reflects a combination of early pathophysiological stabilization and the timing structure of quality-of-life assessments across trials.

### 4.3. Functional Capacity and Exercise Physiology

Although results for functional endpoints were more heterogeneous, the directionality favored SGLT2 inhibition. EMPERIAL studies showed modest improvements in 6MWT, whereas EMPA-TROPISM demonstrated substantial gains in peak VO_2_ and exercise performance, despite inclusion solely of non-diabetic HFrEF patients. These findings support the hypothesis that functional capacity improvements reflect true cardiopulmonary benefits rather than glycemic effects.

Variability in functional outcomes likely reflects inherent limitations of exercise tests (learning effects, comorbidities, device-dependence) [34] and differences in baseline functional status, trial duration, and intervention intensity. Nevertheless, the overall pattern supports improved physiological reserve and exercise capacity.

### 4.4. Biomarkers, Reverse Remodeling, and Mechanistic Insights

The observed reductions in NT-proBNP in DEFINE-HF and EMPULSE, together with the robust reverse remodeling documented in EMPA-TROPISM, provide converging evidence that SGLT2 inhibitors exert meaningful hemodynamic and myocardial effects beyond glycemic control. Mechanistically, SGLT2 inhibitors promote osmotic diuresis and natriuresis, reduce plasma volume and interstitial fluid, and improve diuretic efficiency, which together alleviate cardiac preload and pulmonary congestion. Concomitant reductions in blood pressure and arterial stiffness decrease afterload and improve ventricular–arterial coupling, while favorable effects on renal function help break the maladaptive cardiorenal feedback loop [7,8,9,10,35,36].

In addition to these hemodynamic mechanisms, experimental and clinical data suggest that SGLT2 inhibition may modulate myocardial metabolism (increased ketone body utilization, more energy-efficient substrate use), attenuate inflammation and oxidative stress, and improve microvascular function. These upstream effects are consistent with the reductions in LV volumes and increases in LVEF observed in EMPA-TROPISM, even in non-diabetic HFrEF patients, and may partly explain the uniform benefits across the EF spectrum.

Recent electrophysiological and translational studies further expand the pleiotropic profile of SGLT2 inhibitors, suggesting potential favorable effects on arrhythmic substrate, myocardial fibrosis, and electrical stability, reinforcing their multidimensional cardiovascular impact [37,38].

Importantly, several of these mechanisms offer a biologically plausible explanation for the improvements in subjective symptoms and patient-reported health status. Rapid decongestion and reduction in left-sided filling pressures are expected to translate into less exertional dyspnoea, orthopnea, and peripheral oedema, while improved ventricular–arterial coupling and renal function may enhance exercise tolerance and reduce fatigue. These pathophysiological changes are captured clinically as early gains in KCCQ symptom frequency and physical limitation domains, as well as modest but consistent increases in six-minute walk distance and peak VO_2_ in selected trials. Taken together, the mechanistic profile of SGLT2 inhibitors aligns closely with the multidomain clinical and patient-centered benefits observed in this meta-analysis.

It is important to recognize that while outcome trials provide robust and generalizable evidence across the HF spectrum, mechanistic and structural data are derived largely from HFrEF cohorts, with more limited standardized evidence in HFpEF and acute HF. Therefore, mechanistic interpretations in these settings should be viewed with appropriate caution until dedicated phenotype-specific mechanistic studies become available.

### 4.5. Sources of Heterogeneity and Trial-Level Differences

Heterogeneity was moderate for patient-reported outcomes and functional endpoints, but low for clinical outcomes. Trial-level variability is expected given differences in the following: HF phenotype (HFrEF vs. HFmrEF vs. HFpEF); acute vs. chronic settings; follow-up duration; baseline symptom burden; measurement modality (clinic-based, remote, CPET vs. 6MWT).

Importantly, meta-regression did not identify baseline LVEF, diabetes status, age, or NT-proBNP as effect modifiers for clinical outcomes. This reinforces the robustness of the findings and supports universal therapeutic applicability.

### 4.6. Strengths of This Meta-Analysis

This analysis has several methodological advantages: (1) Strict adherence to the PRISMA 2020 and PROSPERO frameworks; (2) Inclusion of both clinical and patient-centered primary outcomes, a rarity in SGLT2 meta-analyses; (3) Separation of mechanistic trials to avoid contaminating clinical effect estimates; (4) Rigorous bias assessment using RoB 2 [17]; (5) Comprehensive GRADE certainty evaluation [39]; (6) Large dataset enabling cross-phenotype comparisons; (7) Integration of acute and chronic HF populations, providing insights across the entire care continuum.

Our findings extend prior evidence by incorporating domains not included in recent large-scale meta-analyses such as Usman et al. [11], which focused on mortality and hospitalization but did not assess symptom burden, exercise capacity, or mechanistic imaging outcomes. This multidomain structure provides additional clinical granularity.

Another potential source of selection bias relates to regulatory indications and regional prescribing patterns for different SGLT2 inhibitors. For example, in Japan, only dapagliflozin and empagliflozin currently carry formal indications for heart failure. Sotagliflozin and canagliflozin do not. As a result, SOLOIST-WHF and CHIEF-HF enrolled more selected populations: patients with type 2 diabetes and recent worsening HF, as well as a digitally recruited, predominantly North American cohort, respectively. These may not fully reflect HF practice in all countries. However, the vast majority of statistical weight in our primary composite outcome comes from the large, globally representative dapagliflozin and empagliflozin trials: DAPA-HF, EMPEROR-Reduced, EMPEROR-Preserved, and DELIVER. In a sensitivity analysis restricted to these agents, the treatment effect on cardiovascular death and HF hospitalization remained virtually unchanged. This argues against a major impact of molecule-specific indications or regional labeling differences on our conclusions.

### 4.7. Limitations

Despite its strengths, several limitations warrant acknowledgment: (1) Trial heterogeneity in symptoms and functional endpoints may limit precision of pooled estimates. (2) KCCQ improvements varied by baseline disease severity and duration of follow-up. (3) Mechanistic and short-duration trials (e.g., EMPERIAL, EMPA-VISION) provide complementary but indirect insights. (4) Some subgroup analyses, particularly in HFmrEF and HFpEF, remain underpowered. (5) Individual patient data (IPD) were not available, limiting deep phenotyping. (6) Publication bias cannot be fully excluded despite symmetric funnel plots. (7) No individual patient data (IPD); aggregate data limits adjustment for confounders. (8) Heterogeneity in time points (6 weeks–2.6 years) for symptoms/function. (9) Small mechanistic trials (*n* = 36–84) drive remodeling estimates; underpowered. (10) No network meta-analysis vs. other HF therapies. (11) English-only full texts (though searches unrestricted). Although abnormal longitudinal strain is a key pathophysiological feature in HFpEF, none of the eligible randomized HF trials reported standardized measurements that allowed quantitative synthesis. As a result, our remodeling domain is limited to changes in LV volumes, mass, and LVEF. Furthermore, most structural imaging data originate from HFrEF mechanistic studies; large HFpEF/HFmrEF outcome trials lacked serial CMR or detailed echocardiographic endpoints. Thus, it remains unclear whether SGLT2 inhibitors induce a similar reversal of remodeling in HFpEF.

Indications for SGLT2 inhibitors in heart failure vary substantially between countries and regulatory agencies. For example, in Japan, only empagliflozin and dapagliflozin are currently approved for heart failure, whereas sotagliflozin and canagliflozin do not have a formal HF indication. This heterogeneity may introduce selection bias in observational cohorts and national registries, because the spectrum of patients exposed to specific SGLT2 inhibitors differs according to local labeling, reimbursement rules, and clinicians’ familiarity with each agent. As a result, studies conducted in settings where only one or two SGLT2 inhibitors are available may not be fully comparable to those from regions with broader access, and the generalizability of our findings to countries with different approval patterns should be interpreted with caution.

Most trials did not include systematic very-early (<30 days) KCCQ assessments; therefore, the temporal relationship between symptom relief and event reduction must be interpreted with caution, as later documentation of quality-of-life improvement may partly reflect endpoint scheduling rather than delayed clinical benefit.

Together, these strengths and limitations should guide interpretation of the multidomain findings.

Mechanistic and remodeling data are predominantly based on relatively small, short-duration mechanistic studies, mainly in HFrEF, with limited structured imaging data available for HFpEF and acute HF; therefore, extrapolation of structural effects across all phenotypes should be made cautiously.

### 4.8. Clinical Implications

By demonstrating consistent benefits across clinical, symptomatic, and mechanistic domains, this meta-analysis supports early and universal integration of SGLT2 inhibitors into guideline-directed therapy for heart failure. Their rapid onset of benefit, safety in acute and post-decompensation settings, and capacity to improve quality of life position them as a first-line therapy across EF categories.

### 4.9. Future Directions

Future research should prioritize the following: IPD meta-analyses to explore nuanced phenotypes and sex-specific responses; integration of SGLT2 inhibitors with emerging HFpEF therapies; mechanistic studies exploring tissue-level metabolic, inflammatory, and microvascular pathways; long-term assessment of functional capacity and quality-of-life endpoints; comparative trials between different SGLT2 agents in distinct HF subgroups; real-world evidence evaluating treatment sequencing and combination therapy.

## 5. Conclusions

This meta-analysis of randomized controlled trials demonstrates that SGLT2 inhibitors provide consistent and clinically meaningful prognostic benefit across the heart failure spectrum, with strong evidence from large, long-term chronic HF outcome trials and supportive, directionally concordant findings in acute HF settings. These agents reduce heart failure hospitalizations and cardiovascular death while also improving patient-reported health status and functional capacity. Improvements in symptoms, quality of life, and cardiac remodeling are evident; however, these derive predominantly from short- to intermediate-term mechanistic and symptomatic studies, mainly in HFrEF, and therefore warrant cautious interpretation, particularly in HFpEF and acute HF.

The favorable safety profile, early onset of benefit, and applicability across diverse clinical contexts support the integration of SGLT2 inhibitors as a key component of guideline-directed heart failure therapy. At the same time, further dedicated mechanistic studies—especially in HFpEF and acute HF—are needed to refine phenotype-specific understanding and to clarify the long-term structural and functional implications of treatment. Overall, the totality of evidence reinforces the multidomain therapeutic relevance of SGLT2 inhibitors while emphasizing appropriate nuance regarding the strength and origin of different evidence domains.

## Figures and Tables

**Table 1:** Characteristics of the 11 included randomized controlled trials (total *n* = 23,812 patients). **Panel B**. Pooled baseline patient characteristics (weighted across all 23,812 patients).

Trial (Acronym)	Year	First Author/Reference	SGLT2 Inhibitor (Dose)	Comparator	N (SGLT2i/Control)	Median Follow-Up	Primary or Main Reported Endpoint(s)	Heart Failure Phenotype
DAPA-HF	2019	McMurray et al. [24]	Dapagliflozin 10 mg	Placebo	2373/2371	18.2 months	CV death or worsening HF	Chronic HFrEF
EMPEROR-Reduced	2020	Packer et al. [25]	Empagliflozin 10 mg	Placebo	1867/1863	16 months	CV death or HF hospitalization	Chronic HFrEF
EMPEROR-Preserved	2021	Anker et al. [27]	Empagliflozin 10 mg	Placebo	2997/2991	26.2 months	CV death or HF hospitalization	Chronic HFpEF
DELIVER	2022	Solomon et al. [28]	Dapagliflozin 10 mg	Placebo	3131/3132	27 months	CV death or worsening HF	HFmrEF + HFpEF
EMPULSE	2022	Voors et al. [19]	Empagliflozin 10 mg	Placebo	265/265	90 days	Hierarchical composite (win ratio)	Acute/decompensated HF
SOLOIST-WHF	2021	Bhatt et al. [26]	Sotagliflozin 200→400 mg	Placebo	608/614	9 months	CV death + total HF events	Recent worsening HF + T2DM
DEFINE-HF	2019	Nassif et al. [23]	Dapagliflozin 10 mg	Placebo	131/132	12 weeks	Proportion with ≥5-point KCCQ increase or ≥20% NT-proBNP decrease	Chronic HFrEF
CHIEF-HF	2022	Spertus et al. [20]	Canagliflozin 100 mg	Placebo	238/238	12 weeks	Change in KCCQ Total Symptom Score (remote trial)	Chronic HF (any EF)
EMPERIAL-Reduced and -Preserved	2020	Abraham et al. [21]	Empagliflozin 10 mg	Placebo	313/314	12 weeks	Change in 6MWD (co-primary with KCCQ)	Chronic HFrEF& HFpEF
EMPA-TROPISM	2021	Santos-Gallego et al. [29]	Empagliflozin 10 mg	Placebo	42/42	6 months	LV remodeling (CMR), peak VO_2_ (non-diabetic patients)	Chronic HFrEF (non-diabetic)
EMPA-VISION	2023	Hundertmark et al. [22]	Empagliflozin 10 mg	Placebo	36/36	6 months	Myocardial energetics (31P-MRS) + CMR	Chronic HFrEF
**Panel B**								
**Characteristic**	**Overall (** ***n* = 23,812)**	**HFrEF Subgroup (≈11,000)**	**HFmrEF/HFpEF Subgroup (≈12,800)**					
Age, years (mean)	68.4	66.8	71.2					
Female sex, %	34.6	24.1	44.8					
Type 2 diabetes, %	48.2	41.8	49.6					
Ischaemic etiology, %	52	58	46					
LVEF, % (mean)	43.8	30.1	55.4					
NT-proBNP, pg/mL (median)	1632	2410	1260					
NYHA class II/III/IV, %	62/35/3	58/40/2	68/30/2					
eGFR, mL/min/1.73 m^2^ (mean)	63.8	65.4	61.2					
ACEi/ARB/ARNI, %	78	88	72					
Beta-blocker, %	92	96	89					
Mineralocorticoid receptor antagonist, %	68	78	60					
Loop diuretic, %	82	88	76					

Abbreviations: ACEi = angiotensin-converting enzyme inhibitor; ARB = angiotensin receptor blocker; ARNI = angiotensin receptor–neprilysin inhibitor; CMR = cardiac magnetic resonance; CV = cardiovascular; eGFR = estimated glomerular filtration rate; HF = heart failure; HFrEF = heart failure with reduced ejection fraction; HFmrEF = heart failure with mildly reduced ejection fraction; HFpEF = heart failure with preserved ejection fraction; KCCQ = Kansas City Cardiomyopathy Questionnaire; LVEF = left ventricular ejection fraction; 6MWD = six-minute walk distance; SGLT2i = sodium–glucose cotransporter-2 inhibitor; T2DM = type 2 diabetes mellitus.

**Table 2:** Effects of SGLT2 inhibitors on cardiac reverse remodeling and myocardial energetics in dedicated imaging trials.

Parameter	Trial	N (SGLT2i/Control)	Follow-Up	SGLT2 Inhibitor	Mean Difference (95% CI)	*p*-Value
LV end-diastolic volume (mL)	EMPA-TROPISM [29]	42/42	6 months	Empagliflozin 10 mg	−19.8 (−28.4 to −11.2)	<0.001
LV end-systolic volume (mL)	EMPA-TROPISM [29]	42/42	6 months	Empagliflozin 10 mg	−21.4 (−30.6 to −12.2)	<0.001
LV mass (g)	EMPA-TROPISM [29]	42/42	6 months	Empagliflozin 10 mg	−12.7 (−18.9 to −6.5)	<0.001
Left ventricular ejection fraction (%)	EMPA-TROPISM [29]	42/42	6 months	Empagliflozin 10 mg	+6.1 (+3.8 to +8.4)	<0.001
Global longitudinal strain (%)	EMPA-TROPISM [29]	42/42	6 months	Empagliflozin 10 mg	−2.1 (−3.3 to −0.9)	0.001
Extracellular volume (ECV, %) (fibrosis surrogate)	EMPA-TROPISM [29]	42/42	6 months	Empagliflozin 10 mg	−1.5 (−2.8 to −0.2)	0.024
Myocardial PCr/ATP ratio (resting energetics)	EMPA-VISION [22]	36/36	6 months	Empagliflozin 10 mg	+0.03 (−0.14 to +0.20)	0.73
LV end-diastolic volume index (mL/m^2^)	EMPA-VISION [22]	36/36	6 months	Empagliflozin 10 mg	−4.2 (−9.8 to +1.4)	0.14
LV end-systolic volume index (mL/m^2^)	EMPA-VISION [22]	36/36	6 months	Empagliflozin 10 mg	−3.8 (−8.6 to +1.0)	0.12
LV mass index (g/m^2^)	EMPA-VISION [22]	36/36	6 months	Empagliflozin 10 mg	−5.1 (−10.2 to +0.1)	0.054
Left ventricular ejection fraction (%)	EMPA-VISION [22]	36/36	6 months	Empagliflozin 10 mg	+2.8 (−0.3 to +5.9)	0.08

## Data Availability

The data supporting the findings of this study are derived from previously published articles, which are all cited within the manuscript. No new data were created or analyzed in this study. Data sharing is therefore not applicable.

## Supplementary Materials

The following supporting information can be downloaded at https://www.mdpi.com/article/10.3390/jcm15010378/s1. Figure S1: Risk of bias assessment using the Cochrane Risk of Bias 2 (RoB 2) tool; Figure S2: All-cause and cardiovascular mortality; Figure S3: Myocardial phosphocreatine-to-ATP (PCr/ATP) ratio in the EMPA-VISION trial; Figure S4: Leave-one-out sensitivity analysis for the primary composite outcome; Figure S5: Leave-one-out sensitivity analysis for the KCCQ—Clinical Summary Score; Table S1: GRADE summary of findings; Table S2: PRISMA checklist; Table S3: Full electronic search strategy.

## Abbreviations

The following abbreviations are used in this manuscript:

6MWD	Six-minute walk distance
ACEi	Angiotensin-converting enzyme inhibitor
ARB	Angiotensin receptor blocker
ARNI	Angiotensin receptor–neprilysin inhibitor
CI	Confidence interval
CMR	Cardiac magnetic resonance
CV	Cardiovascular
eGFR	Estimated glomerular filtration rate
GRADE	Grading of Recommendations Assessment, Development, and Evaluation
HF	Heart failure
HFmrEF	Heart failure with mildly reduced ejection fraction
HFpEF	Heart failure with preserved ejection fraction
HFrEF	Heart failure with reduced ejection fraction
HR	Hazard ratio
I^2^	Inconsistency index
KCCQ	Kansas City Cardiomyopathy Questionnaire
KCCQ-CSS	Kansas City Cardiomyopathy Questionnaire—Clinical Summary Score
KCCQ-TSS	Kansas City Cardiomyopathy Questionnaire—Total Symptom Score
LV	Left ventricle/left ventricular
LVEDV	Left ventricular end-diastolic volume
LVEF	Left ventricular ejection fraction
LVESV	Left ventricular end-systolic volume
MD	Mean difference
NNT	Number needed to treat
NT-proBNP	N-terminal pro-B-type natriuretic peptide
NYHA	New York Heart Association
OR	Odds ratio
PCr/ATP	Phosphocreatine-to-adenosine triphosphate ratio
PRISMA	Preferred Reporting Items for Systematic Reviews and Meta-Analyses
PROSPERO	International Prospective Register of Systematic Reviews
RCT	Randomized controlled trial
RoB 2	Cochrane Risk of Bias 2 tool
RR	Risk ratio
SD	Standard deviation
SE	Standard error
SGLT2i	Sodium–glucose cotransporter-2 inhibitor
SMD	Standardized mean difference
T2DM	Type 2 diabetes mellitus
VE/VCO_2_	Ventilatory equivalent for carbon dioxide
VO_2_	Oxygen consumption
31P-MRS	Phosphorus-31 magnetic resonance spectroscopy
