# Supplementary material for: Effects of SGLT2 Inhibitors on Clinical Outcomes, Symptoms, Functional Capacity, and Cardiac Remodeling in Heart Failure: A Comprehensive Systematic Review and Multidomain Meta-Analysis of Randomized Trials
Source: J Clin Med. 2026 Jan 4;15(1):378. doi: 10.3390/jcm15010378 (PMC12787172; doi:10.3390/jcm15010378)
Supplement: Supplementary file 1 [file jcm-15-00378-s001.zip › jcm-4057343-supplementary.pdf]

Supplementary Materials

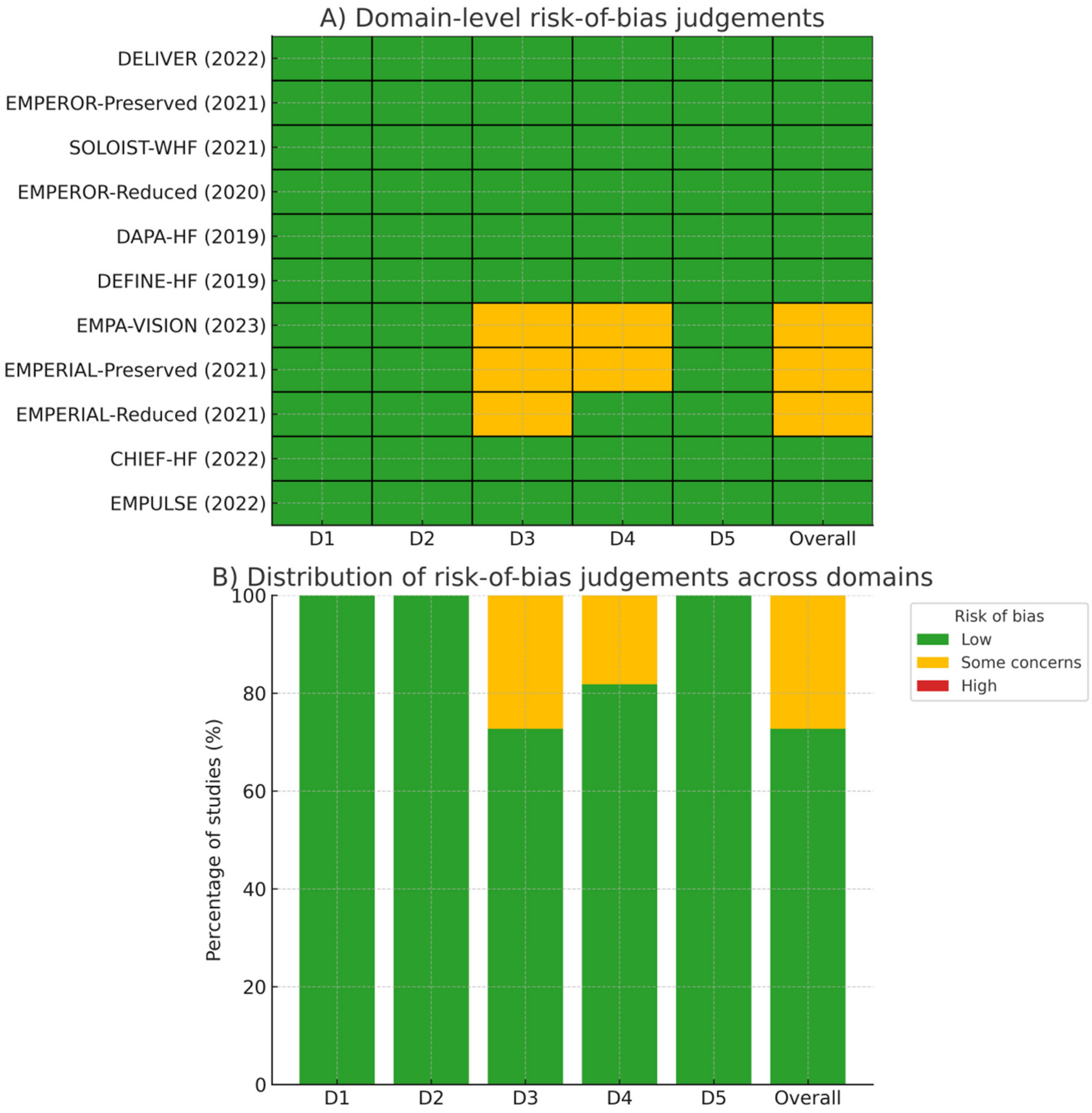

Supplementary Figure S1. Risk of bias assessment using the Cochrane Risk of Bias 2 (RoB 2) tool. (A) Traffic-light plot of domain-level judgements for each included randomized controlled trial. (B) Weighted bar plot of the distribution of risk-of-bias judgements within each domain. Green = low risk; yellow = some concerns; red = high risk. Overall risk of bias is shown in the rightmost column.

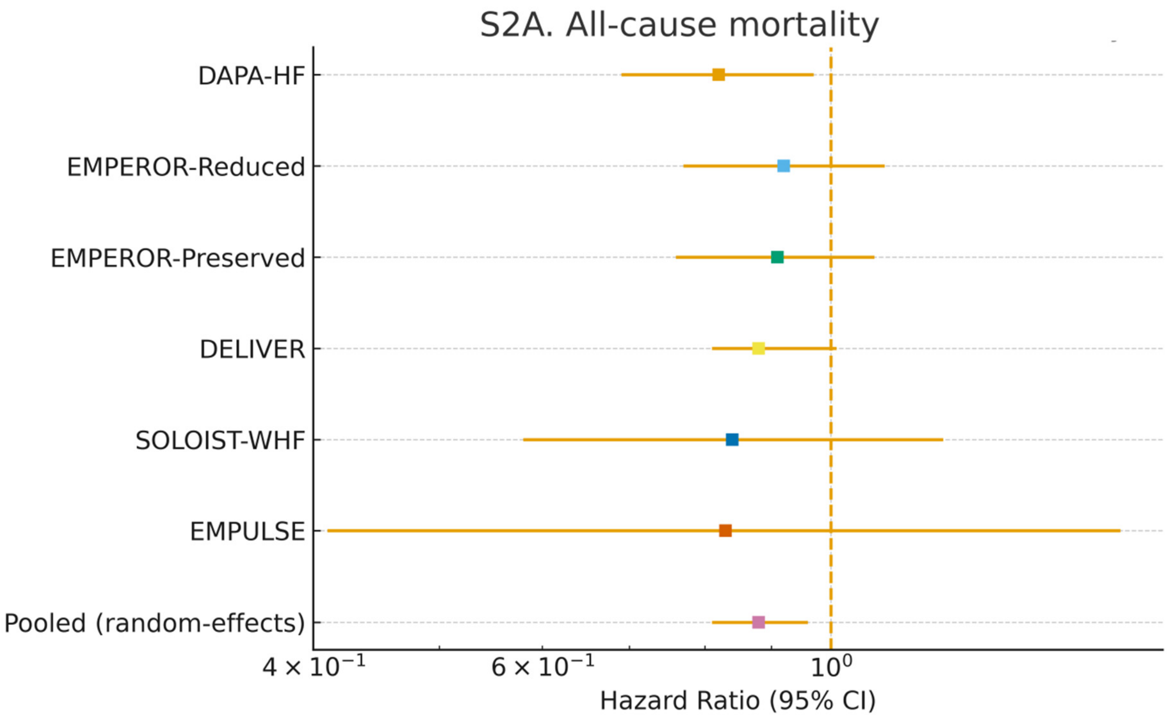

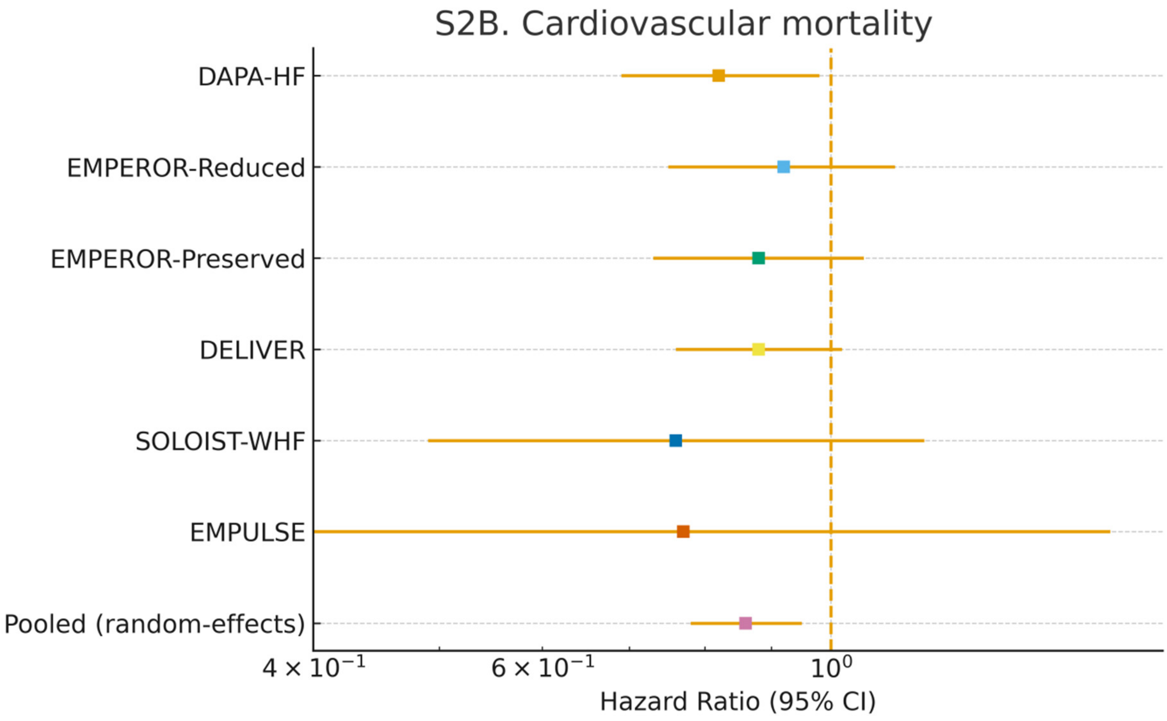

Supplementary Figure S2. All-cause and cardiovascular mortality with SGLT2 inhibitors versus placebo. (A) Forest plot of all-cause mortality across the six major heart failure outcome trials. (B) Forest plot of cardiovascular mortality. Squares represent study-level hazard ratios, horizontal lines indicate 95% confidence intervals, and the pooled random-effects estimates are shown at the bottom of each panel. The vertical dashed line indicates the line of no effect (HR = 1.0).

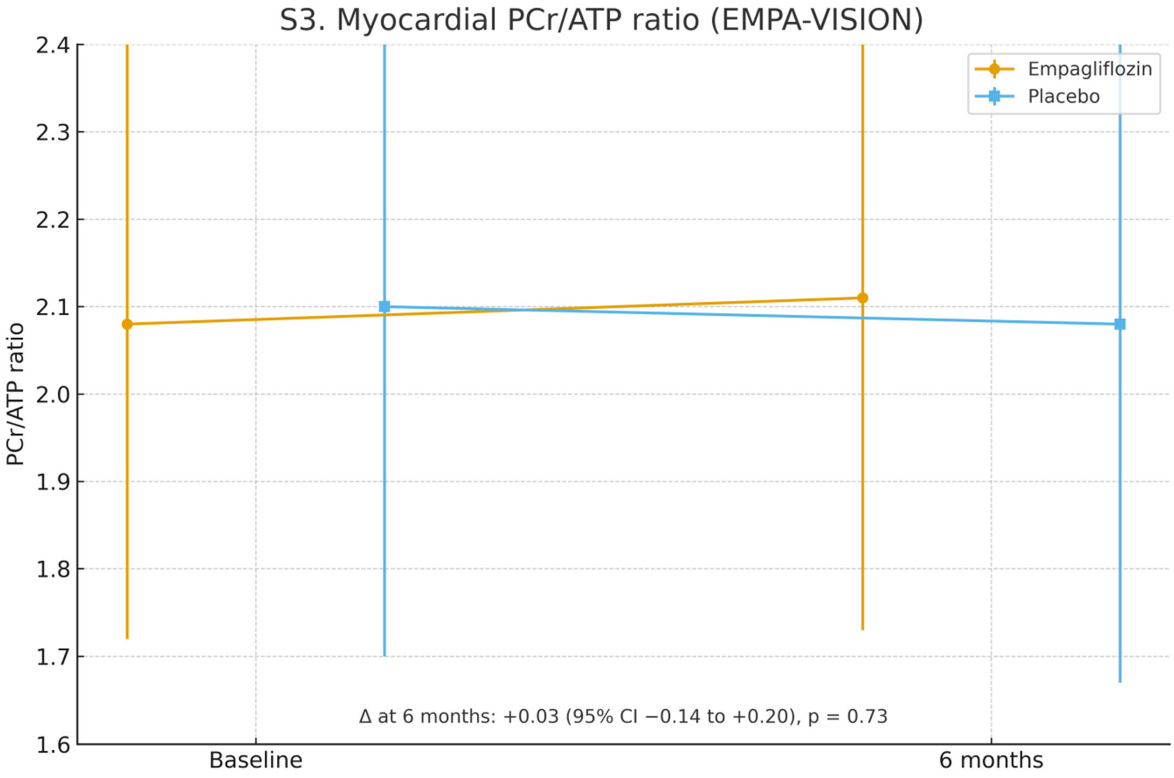

Supplementary Figure S3. Myocardial phosphocreatine-to-ATP (PCr/ATP) ratio assessed by phosphorus-31 magnetic resonance spectroscopy in the EMPA-VISION trial. Mean ± SD values at baseline and 6 months are shown for patients randomized to empagliflozin and placebo. The between-group difference in change from baseline to 6 months was +0.03 (95% CI −0.14 to +0.20; p = 0.73), indicating no significant effect of SGLT2 inhibition on resting myocardial high-energy phosphate metabolism.

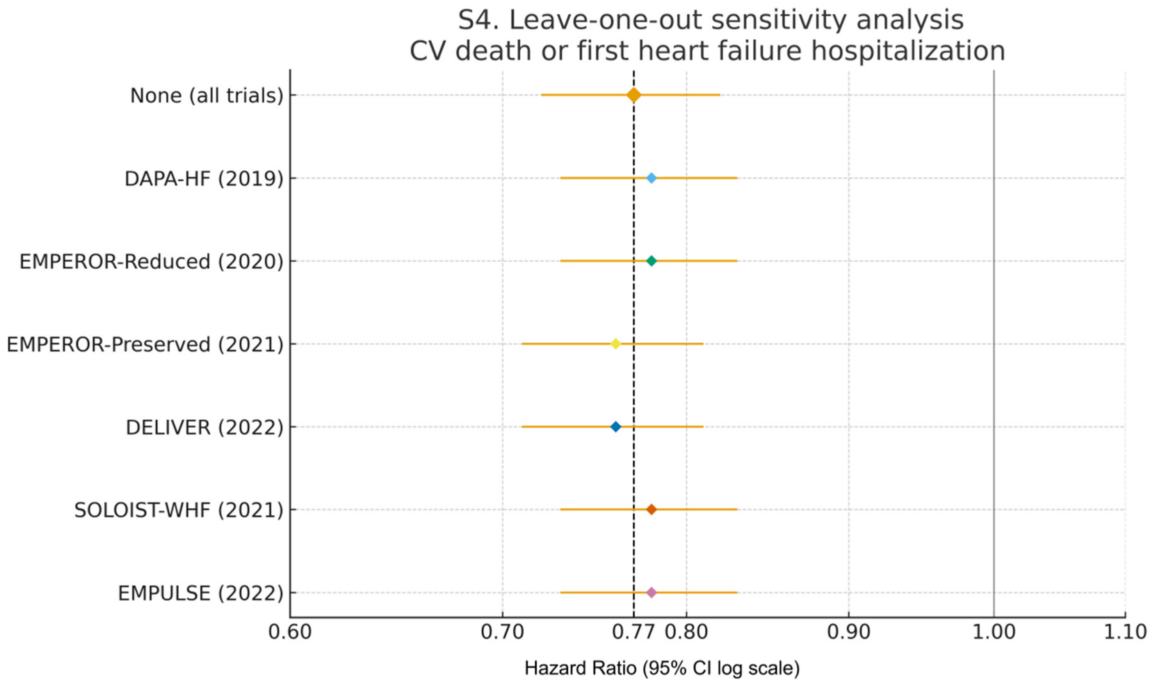

Supplementary Figure S4. Leave-one-out sensitivity analysis for the primary composite outcome (cardiovascular death or first heart failure hospitalization). The vertical dashed line indicates the overall pooled hazard ratio (HR 0.77, 95% CI 0.72–0.82) when all six outcome trials are included. Each diamond represents the pooled HR and 95% CI after exclusion of the respective trial. The overall effect remains robust and consistent across all leave-one-out iterations.

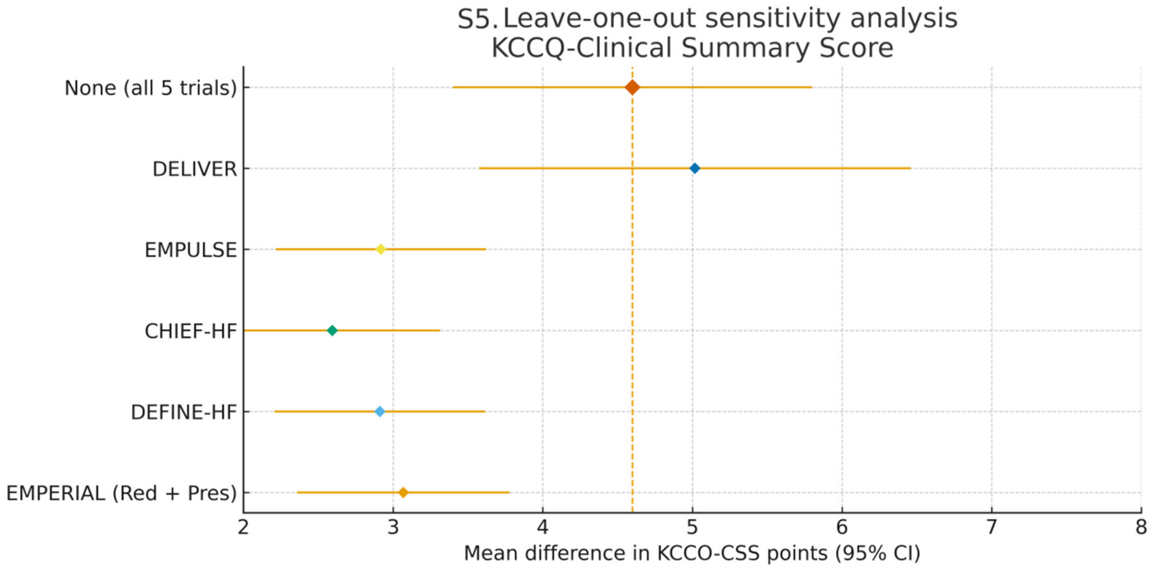

Supplementary Figure S5. Leave-one-out sensitivity analysis for the Kansas City Cardiomyopathy Questionnaire–Clinical Summary Score (KCCQ-CSS). The dashed vertical line indicates the overall pooled mean difference of +4.6 points (95% CI 3.4–5.8) across all five trials. Each diamond represents the pooled mean difference and 95% confidence interval when the respective study is omitted. The treatment effect on KCCQ-CSS remains stable and clinically meaningful across all leave-one-out iterations.

Supplementary Table S1. GRADE summary of findings – Certainty of evidence

Outcome	No. of trials	No. of patients	Effect estimate (95% CI)	I^2 (%)	Certainty (GRADE)	Reason for downgrading
**Clinical outcomes**						
CV death or first HF hospitalization (primary composite)	6	22 927	HR 0.77 (0.72–0.82)	28	High	—
All-cause mortality	6	22 927	HR 0.88 (0.81–0.96)	0	High	—
Cardiovascular mortality	6	22 927	HR 0.86 (0.78–0.95)	12	High	—
Recurrent HF hospitalizations (rate ratio)	4	18 875	Rate ratio 0.71 (0.66–0.76)	0	High	—
**Symptoms and health-related quality of life**						
KCCQ-Clinical Summary	5	8 714	MD +4.6 points	52	Moderate	↓1 Inconsistency

Outcome	No. of trials	No. of patients	Effect estimate (95% CI)	I^2 (%)	Certainty (GRADE)	Reason for downgrading
Score (change from baseline)			(3.4 to 5.8)			(moderate I^2)
Clinically meaningful improvement (≥5-point increase in KCCQ-CSS)	5	8 714	OR 1.49 (1.32–1.68); NNT = 12	31	Moderate	↓1 Inconsistency
**Functional capacity**						
6-minute walk distance	4	1 203	MD +21.8 m (9.4 to 34.2)	61	Moderate	↓1 Inconsistency (substantial I^2)
Peak VO_2 (EMPA-TROPISM trial only)	1	84	MD +1.9 mL/kg/min (0.9 to 2.9)	—	Low	↓1 Indirectness, ↓1 Imprecision (single small trial)
**Cardiac remodeling and energetics**						
LV end-diastolic volume (CMR)	2	156	MD −19.8 mL (−28.4 to −11.2)	0	Moderate	↓1 Imprecision (small total sample size)
LV ejection fraction (CMR)	2	156	MD +6.1% (3.8 to 8.4)	0	Moderate	↓1 Imprecision (small total sample size)
Myocardial PCr/ATP ratio (31P-MRS)	1	72	MD +0.03 (−0.14 to 0.20)	—	Low	↓1 Imprecision, ↓1 Indirectness (single very small trial)

Abbreviations: CI = confidence interval; CMR = cardiac magnetic resonance; CV = cardiovascular; HF = heart failure; HR = hazard ratio; KCCQ = Kansas City Cardiomyopathy Questionnaire; LVEF = left ventricular ejection fraction; MD = mean difference; NNT = number needed to treat; OR = odds ratio; PCr/ATP = phosphocreatine-to-adenosine triphosphate ratio; VO_2 = oxygen consumption.

Supplementary Table S2. PRISMA checklist.

Section and Topic	Item #	Checklist item	Location where item is reported
**TITLE**			
Title	1	Identify the report as a systematic review.	Title (first page) — „Multidomain Effects of SGLT2 Inhibitors in Heart Failure: A Systematic Review and Meta-analysis"
**ABSTRACT**			
Abstract	2	See the PRISMA 2020 for Abstracts checklist.	Structured Abstract — PRISMA 2020 for Abstracts

Section and Topic	Item #	Checklist item	Location where item is reported
			checklist (entire abstract section).
**INTRODUCTION**			
Rationale	3	Describe the rationale for the review in the context of existing knowledge.	Introduction, paragraphs 1–3
Objectives	4	Provide an explicit statement of the objective(s) or question(s) the review addresses.	Introduction, last paragraph: explicit objective statement
**METHODS**			
Eligibility criteria	5	Specify the inclusion and exclusion criteria for the review and how studies were grouped for the syntheses.	Methods → Eligibility Criteria
Information sources	6	Specify all databases, registers, websites, organisations, reference lists and other sources searched or consulted to identify studies. Specify the date when each source was last searched or consulted.	Methods → Data Sources and Search Strategy (first paragraph)
Search strategy	7	Present the full search strategies for all databases, registers and websites, including any filters and limits used.	Supplementary Table S3 — Full Search Strategies
Selection process	8	Specify the methods used to decide whether a study met the inclusion criteria of the review, including how many reviewers screened each record and each report retrieved, whether they worked independently, and if applicable, details of automation tools used in the process.	Methods → Study Selection (screening by two independent reviewers using predefined criteria)
Data collection process	9	Specify the methods used to collect data from reports, including how many reviewers collected data from each report, whether they worked independently, any processes for obtaining or confirming data from study investigators, and if applicable, details of automation tools used in the process.	Methods → Data Extraction (two reviewers extracted independently; discrepancies resolved by consensus)
Data items	10a	List and define all outcomes for which data were sought. Specify whether all results that were compatible with each outcome domain in each study were sought (e.g. for all measures, time points, analyses), and if not, the methods used to decide which results to collect.	Methods → Outcomes and Definitions
	10b	List and define all other variables for which data were sought (e.g. participant and intervention characteristics, funding sources).	Methods → Data Extraction (“study

**Section and Topic**	**Item #**	**Checklist item**	**Location where item is reported**
		Describe any assumptions made about any missing or unclear information.	characteristics, patient characteristics, HF phenotype, intervention, comparator…”)
Study risk of bias assessment	11	Specify the methods used to assess risk of bias in the included studies, including details of the tool(s) used, how many reviewers assessed each study and whether they worked independently, and if applicable, details of automation tools used in the process.	Methods → Risk of Bias Assessment (RoB 2, two reviewers independently)
Effect measures	12	Specify for each outcome the effect measure(s) (e.g. risk ratio, mean difference) used in the synthesis or presentation of results.	Methods → Data Synthesis (“hazard ratios for event outcomes; mean differences for continuous outcomes”)
Synthesis methods	13a	Describe the processes used to decide which studies were eligible for each synthesis (e.g. tabulating the study intervention characteristics and comparing against the planned groups for each synthesis (item #5)).	Methods → Data Synthesis (how trials were grouped into four domains: clinical, symptoms, functional, energetics)
	13b	Describe any methods required to prepare the data for presentation or synthesis, such as handling of missing summary statistics, or data conversions.	Methods → Data Preparation (conversion of 95% CI → SE, handling missing SDs using validated methods)
	13c	Describe any methods used to tabulate or visually display results of individual studies and syntheses.	Methods → Statistical Analysis (forest plots, tables, structured presentation)
	13d	Describe any methods used to synthesize results and provide a rationale for the choice(s). If meta-analysis was performed, describe the model(s), method(s) to identify the presence and extent of statistical heterogeneity, and software package(s) used.	Methods → Statistical Analysis (random-effects, Hartung–Knapp, Paule–Mandel τ^2, heterogeneity via I^2

Section and Topic	Item #	Checklist item	Location where item is reported
			+ prediction intervals)
	13e	Describe any methods used to explore possible causes of heterogeneity among study results (e.g. subgroup analysis, meta-regression).	Methods → Exploratory analyses (subgroup analyses by HF phenotype, hospitalization strata when available)
	13f	Describe any sensitivity analyses conducted to assess robustness of the synthesized results.	Methods → Sensitivity analyses + Supplementary Figures S4–S5
Reporting bias assessment	14	Describe any methods used to assess risk of bias due to missing results in a synthesis (arising from reporting biases).	Methods → Risk of Bias Due to Missing Results + Supplementary Figure S5 funnel plots
Certainty assessment	15	Describe any methods used to assess certainty (or confidence) in the body of evidence for an outcome.	Methods → GRADE Approach + Supplementary Table S1 (GRADE Evidence Summary)
**RESULTS**			
Study selection	16a	Describe the results of the search and selection process, from the number of records identified in the search to the number of studies included in the review, ideally using a flow diagram.	Results → Study Selection + Figure 1 (PRISMA flow diagram)
	16b	Cite studies that might appear to meet the inclusion criteria, but which were excluded, and explain why they were excluded.	Results → Study Selection (exclusion reasons paragraph)
Study characteristics	17	Cite each included study and present its characteristics.	Results → Study Characteristics + Table 1
Risk of bias in studies	18	Present assessments of risk of bias for each included study.	Results → Risk of Bias + Supplementary

Section and Topic	Item #	Checklist item	Location where item is reported
			Figure S1
Results of individual studies	19	For all outcomes, present, for each study: (a) summary statistics for each group (where appropriate) and (b) an effect estimate and its precision (e.g. confidence/credible interval), ideally using structured tables or plots.	Results → Outcome-specific paragraphs + Figures 2–5 + numeric values in-text
Results of syntheses	20a	For each synthesis, briefly summarise the characteristics and risk of bias among contributing studies.	Results → Domain-level summaries (beginning of each subsection)
	20b	Present results of all statistical syntheses conducted. If meta-analysis was done, present for each the summary estimate and its precision (e.g. confidence/credible interval) and measures of statistical heterogeneity. If comparing groups, describe the direction of the effect.	Results → Pooled effects + Figures 2–5 (HR, MD, I^2, τ^2)
	20c	Present results of all investigations of possible causes of heterogeneity among study results.	Results → Heterogeneity exploration (subgroups in acute HF, EF phenotypes)
	20d	Present results of all sensitivity analyses conducted to assess the robustness of the synthesized results.	Results → Sensitivity analyses + Supplementary Figures S4–S5
Reporting biases	21	Present assessments of risk of bias due to missing results (arising from reporting biases) for each synthesis assessed.	Supplementary Figure S5 (funnel plots) + Results → Bias assessment paragraph
Certainty of evidence	22	Present assessments of certainty (or confidence) in the body of evidence for each outcome assessed.	Supplementary Table S1 (GRADE)
**DISCUSSION**			
Discussion	23a	Provide a general interpretation of the results in the context of other evidence.	Discussion → First paragraph (overall interpretation)
	23b	Discuss any limitations of the evidence included in the review.	Discussion → Limitations of evidence

Section and Topic	Item #	Checklist item	Location where item is reported
			(paragraph 2)
	23c	Discuss any limitations of the review processes used.	Discussion → Methodological limitations (paragraph 3)
	23d	Discuss implications of the results for practice, policy, and future research.	Discussion → Implications (final paragraph)
**OTHER INFORMATION**			
Registration and protocol	24a	Provide registration information for the review, including register name and registration number, or state that the review was not registered.	Methods → PROSPERO (CRD420251235850)
	24b	Indicate where the review protocol can be accessed, or state that a protocol was not prepared.	No separate protocol was prepared beyond PROSPERO registration.
	24c	Describe and explain any amendments to information provided at registration or in the protocol.	No amendments were made.
Support	25	Describe sources of financial or non-financial support for the review, and the role of the funders or sponsors in the review.	Funding Statement ("Supported by Victor Babeș University of Medicine and Pharmacy Timișoara…")
Competing interests	26	Declare any competing interests of review authors.	Conflicts of Interest Statement ("The authors declare no conflict of interest.")
Availability of data, code and other materials	27	Report which of the following are publicly available and where they can be found: template data collection forms; data extracted from included studies; data used for all analyses; analytic code; any other materials used in the review.	All extracted data, analytic code, and supplementary figures are available upon request; search strategies and RoB/GRADE tables

Section and Topic	Item #	Checklist item	Location where item is reported
			are provided in Supplementary Material.

Supplementary Table S3. Full electronic search strategy for each database (run on 1 February 2025)

Database	Date of search	Platform / URL	Search strategy (exact string copied into the search box)	Hits
**PubMed / MEDLINE**	1 February 2025	https://pubmed.ncbi.nlm.nih.gov	("SGLT2 inhibitor*" OR "SGLT-2 inhibitor*" OR dapagliflozin OR empagliflozin OR canagliflozin OR sotagliflozin OR ertugliflozin OR bexagliflozin OR "sodium-glucose cotransporter 2") AND ("heart failure" OR "cardiac failure" OR "heart decompensation" OR HFrEF OR HFmrEF OR HFpEF OR "preserved ejection fraction" OR "reduced ejection fraction" OR "myocardial failure") AND (randomized OR randomised OR "randomised controlled trial" OR "randomized controlled trial" OR RCT OR "clinical trial" OR placebo) Filters: Humans, English OR other languages, 2010/01/01 to 2025/02/01	2 184
**Embase (Ovid)**	1 February 2025	https://ovidsp.ovid.com	1. (SGLT2 inhibitor* or SGLT-2 inhibitor* or dapagliflozin or empagliflozin or canagliflozin or sotagliflozin or ertugliflozin or bex).af. 2. (heart failure or cardiac failure or heart decompensation or HFrEF or HFmrEF or HFpEF or preserved ejection fraction or reduced ejection fraction).af. 3. (randomized controlled trial or randomised controlled trial or RCT or placebo).pt. 4. 1 and 2 and 3 5. limit 4 to yr="2010 -Current" 6. limit 5 to (human and (embryonic or infant or child or adolescent or adult or "middle age" or "all aged" or "aged"))	3 412
**Cochrane CENTRAL**	1 February 2025	https://www.cochranelibrary.com/central	#1 MeSH descriptor: [Sodium-Glucose Transporter 2 Inhibitors] explode all trees #2 (SGLT2 or SGLT-2):ti,ab,kw #3 (dapagliflozin or empagliflozin or	892

**Database**	**Date of search**	**Platform / URL**	**Search strategy (exact string copied into the search box)**	**Hits**
			canagliflozin or sotagliflozin or Forxiga or Jardiance or Invokana):ti,ab,kw #4 MeSH descriptor: [Heart Failure] explode all trees #5 ("heart failure" or "cardiac failure" or HFrEF or HFpEF or HFmrEF):ti,ab,kw #6 #1 or #2 or #3 #7 #4 or #5 #8 #6 and #7 #9 (Trials)	
**Web of Science Core Collection**	1 February 2025	https://www.webofscience.com	TS=((SGLT2 OR "SGLT-2" OR dapagliflozin OR empagliflozin OR canagliflozin OR sotagliflozin) AND ("heart failure" OR HFrEF OR HFmrEF OR HFpEF OR "cardiac failure") AND (randomized OR randomised OR RCT OR "clinical trial" OR placebo)) Timespan: 2010–2025 Indexes: SCI-EXPANDED, SSCI, A&HCI, CPCI-S, CPCI-SSH, ESCI	2 254
**Total before deduplication**				**9 742**
**Total after deduplication**				**8 742**
